# IKKα/CHUK Regulates Extracellular Matrix Remodeling Independent of Its Kinase Activity to Facilitate Articular Chondrocyte Differentiation

**DOI:** 10.1371/journal.pone.0073024

**Published:** 2013-09-02

**Authors:** Eleonora Olivotto, Miguel Otero, Annalisa Astolfi, Daniela Platano, Annalisa Facchini, Stefania Pagani, Flavio Flamigni, Andrea Facchini, Mary B. Goldring, Rosa Maria Borzì, Kenneth B. Marcu

**Affiliations:** 1 Laboratory of Immunorheumatology and Tissue Regeneration/RAMSES, Rizzoli Orthopedic Research Institute, Bologna, Italy; 2 Research Division, Hospital for Special Surgery and Weill Cornell Medical College, New York, New York, United States of America; 3 “G.Prodi” Cancer Research Center, University of Bologna, Bologna, Italy; 4 Medical and Surgical Sciences Department, University of Bologna, Bologna, Italy; 5 Biomedical and Neuromotor Sciences Department, University of Bologna, Bologna, Italy; 6 Biochemistry and Cell Biology Department, Stony Brook University, Stony Brook, New York, United States of America; UMR CNRS 5242 - ENS de Lyon- Université Lyon 1, France

## Abstract

**Background:**

The non-canonical NF-κB activating kinase IKKα, encoded by *CHUK* (conserved-helix-loop-helix-ubiquitous-kinase), has been reported to modulate pro- or anti- inflammatory responses, cellular survival and cellular differentiation. Here, we have investigated the mechanism of action of IKKα as a novel effector of human and murine chondrocyte extracellular matrix (ECM) homeostasis and differentiation towards hypertrophy.

**Methodology/Principal Findings:**

IKKα expression was ablated in primary human osteoarthritic (OA) chondrocytes and in immature murine articular chondrocytes (iMACs) derived from *IKKα^f/f^:CreERT2* mice by retroviral-mediated stable shRNA transduction and Cre recombinase-dependent Lox P site recombination, respectively. MMP-10 was identified as a major target of IKKα in chondrocytes by mRNA profiling, quantitative RT-PCR analysis, immunohistochemistry and immunoblotting. ECM integrity, as assessed by type II collagen (COL2) deposition and the lack of MMP-dependent COL2 degradation products, was enhanced by IKKα ablation in mice. MMP-13 and total collagenase activities were significantly reduced, while TIMP-3 (tissue inhibitor of metalloproteinase-3) protein levels were enhanced in IKKα-deficient chondrocytes. IKKα deficiency suppressed chondrocyte differentiation, as shown by the quantitative inhibition of.Alizarin red staining and the reduced expression of multiple chondrocyte differentiation effectors, including Runx2, Col10a1 and Vegfa,. Importantly, the differentiation of IKKα-deficient chondrocytes was rescued by a kinase-dead IKKα protein mutant.

**Conclusions/Significance:**

IKKα acts independent of its kinase activity to help drive chondrocyte differentiation towards a hypertrophic-like state. IKKα positively modulates ECM remodeling via multiple downstream targets (including MMP-10 and TIMP-3 at the mRNA and post-transcriptional levels, respectively) to maintain maximal MMP-13 activity, which is required for ECM remodeling leading to chondrocyte differentiation. Chondrocytes are the unique cell component in articular cartilage, which are quiescent and maintain ECM integrity during tissue homeostasis. In OA, chondrocytes reacquire the capacity to proliferate and differentiate and their activation results in pronounced cartilage degeneration. Τηυσ, our findings are also of potential relevance for defining the onset and/or progression of OA disease.

## Introduction

Cells differentiate in response to environmental signals from their neighbors and also from extracellular matrix (ECM) effectors. ECM proteins directly and indirectly modulate signal transduction pathways triggered by growth and differentiation factors [1]. Since ECM structural changes driven by enzyme-mediated remodeling impact on cell differentiation cues, a thorough understanding of the mechanisms that control ECM formation and stability is of critical importance for defining the differentiation of a variety of tissues during mammalian development after birth and throughout adult life [1]. ECM remodeling is mediated by a large number of enzymes and the family of matrix metalloproteinases (MMPs) plays critical roles in this process [1].

Chondrocytes differentiating from mesenchymal progenitors have essential roles in cartilage formation and homeostasis and in skeletal development by synthesizing the templates, or cartilage anlagen, in a process termed chondrogenesis that results in limb formation [2]. After mesenchymal condensation and chondroprogenitor differentiation, chondrocytes proliferate, produce an elaborate ECM, terminally differentiate to hypertrophy, and then succumb to programmed cell death (PCD); the replacement of hypertrophic cartilage by bone culminates this process called endochondral ossification [2]. After birth a somewhat analogous chondrocyte differentiation process occurs in the postnatal growth plate, driving rapid skeletal growth [3]. During endochondral ossification hypertrophic chondrocytes undergo dramatic, stress-associated ECM remodeling, which has also been suggested as a “developmental model” to grasp the contributions of exacerbated environmental stresses in the onset and progression of osteoarthritis (OA) [4]–[9]. This concept is supported by findings in early OA cartilage lesions revealing up-regulation of chondrocyte differentiation-related genes, and by *in vivo* studies showing that alterations in ECM structural integrity or in effectors of progression to hypertrophy can lead to OA pathology [10]. Indeed, alterations in the mineral content and thickness of calcified cartilage, tidemark advancement and enhanced expression of COL10A1 (type×collagen protein), MMP-13 and Runx2 all occur in the context of OA disease to varying degrees, and simulate a recapitulation of chondrocyte differentiation towards a hypertrophic-like phenotype [9], [11]–[14]. The Sox9 and Runx2 transcriptional activators function in an interrelated and stepwise manner at the early stages of chondrocyte differentiation and subsequent hypertrophic maturation [15]. Sox9 and Runx2 expression profiles oppose each other during chondrogenesis and terminal chondrocyte differentiation with the exception of the window between periarticular and proliferating chondrocytes [2]. Sox9 is essential for chondrocyte specification and early differentiation: and it also delays hypertrophic differentiation by controlling Runx2 expression and β-catenin signaling [16], which helps to maintain chondrocytes in an arrested state prior to the onset of hypertrophy. Runx2 instead drives chondrocyte hypertrophy prior to endochondral ossification {reviewed in [2]}. In addition, pro-inflammatory activation of the canonical NF-κB pathway was reported to inhibit mesenchymal chondrocytic differentiation through the transcriptional and post-transcriptional down-modulation of Sox9 mRNA [17], [18], and NF-κB activation was also found to facilitate osteogenesis via BMP (bone morphogenic protein)-mediated induction of Runx2 [19]. Thus, due to the differential effects of Sox9 and Runx2 on chondrogenic differentiation, chronic NF-κB activation in OA chondrocytes could inhibit the early pre-hypertrophic phase, while simultaneously promoting the terminal hypertrophic phase and thereby contributing to abnormal ECM remodeling. ELF3, an epithelial cell-specific ETS family transcription factor, is also subject to NF-κB-dependent IL-1β signaling in chondrocytes wherein it suppresses *COL2A1* gene expression [20] and directly activates *MMP13* transcription [21]. Another direct target of the canonical NF-κB signaling, HIF2α, plays a central role in OA cartilage, where it interconnects inflammatory ECM degradative processes with chondrocyte hypertrophy, controlling the expression of MMP13, NOS2 and VEGF among other factors [22], [23]. Thus, elevated canonical NF-κB signaling in OA vs. normal articular chondrocytes (ACs) could contribute to cartilage degeneration in OA by affecting a number of downstream processes, particularly in response to extrinsic stress/inflammatory signals.

MMP-13 (collagenase 3) becomes involved in the process of chondrocyte ECM remodeling in the late hypertrophic zone of growth plates prior to endochondral ossification, and is also the major type II collagen-degrading enzyme that drives erosion of the cartilage collagen network in OA disease [9], [24]–[27]. Conditional ablations of *Mmp13* in murine chondrocytes and osteoblasts have shown that cartilage ECM remodeling is essential in controlling chondrocyte terminal differentiation to hypertrophy and subsequent apoptosis, as well as angiogenesis and osteoblast recruitment [28]. Moreover, global *Mmp13* gene knockout (KO) as well as conditional cartilage-specific *Mmp13* KO mice are protected against post-traumatic surgically induced OA [27], [29]. Normally, articular chondrocytes (ACs) *in vivo* express low levels of MMP-13; but in sharp contrast, OA chondrocytes display enhanced levels and activities of MMP-13 and other matrix-degrading enzymes, which in part stem from the activated state of OA chondrocytes in response to abnormal stress and inflammatory signals {reviewed in [30]}. *Mmp13* transcriptional control depends on several key transcription factors, including ELF3, Runx2, AP-1 (cFos/cJun), p130^cas^ nuclear matrix transcription factor 4 (NMP-4), and the canonical NF-κB subunits p65/p50 [21], [31]–[34]. MMP-13, like most MMPs, is secreted in an inactive pro-form that is cleaved by specific proteases (including MMP-14, MMP-2 and MMP-10) to produce the active enzyme [35]–[37], and its activity is negatively controlled by tissue inhibitors of metalloproteinases (TIMPs) [38]. Thus, MMP-13 is subject to tight regulation at the transcriptional and post-translational levels, and a more thorough understanding of the regulatory factors that impact on its control in chondrocytes remains important.

We previously demonstrated that chondrocyte ECM remodeling and differentiation *in vitro* depends on MMP-13 [39] and on the NF-κB activating kinases IKKα and IKKβ [40]. Micromass and analogous 3-dimensional (3D) pellet cultures of primary articular chondrocytes (ACs) show “phenotypic plasticity” by recapitulating aspects of terminal differentiation to hypertrophy akin to that of mesenchymal stem cells undergoing chondrogenesis [41]. We found that ablating expression of either MMP-13 or IKKα in 3D differentiating cultures of human ACs dramatically stabilized their ECM, enhanced cell viability and strongly suppressed their differentiation towards a hypertrophic-like state [39], [40]. IKKα KD articular chondrocytes (ACs) were unable to differentiate to a terminal, hypertrophic-like state [40]. Importantly, IKKα loss did not alter the level of MMP-13 protein but appeared to inhibit collagenase activity, as revealed by the pronounced suppression of type II collagen 3/4C neo-epitope fragments (COL2–3/4C) in IKKα KD pellet cultures [40]. Interestingly, the presence of MMP-13 and IKKα in differentiating chondrocytes impacted on the subcellular localization of Sox9 [39], with Sox9 largely displaying a peri-nuclear staining pattern in differentiating wild type (WT) chondrocyte pellets but instead localizing within the nuclei of chondrocytes lacking MMP-13 or IKKα [39]. Previous studies showed that Sox9 inhibits β-catenin-dependent signaling in chondrocytes [42] and other cell types [43]; and in accord with this earlier work, we showed an inverse correlation of Sox9 nuclear localization with β-catenin stability and activation status [39].

IKKα (also called IKK1 and encoded by the gene *CHUK* (conserved-helix-loop-helix-ubiquitous kinase) is a stoichiometric component of the NF-κB activating IKK signalosome complex; and it also has a variety of important functions not associated with NF-κB signaling. IKKα/CHUK was originally discovered as a ubiquitously expressed, evolutionarily conserved protein with a carboxy-terminal helix-loop-helix and amino terminal serine-threonine kinase domains [44]. IKKα/CHUK normally resides in the cytoplasmic IKK complex with the related kinase IKKβ (also called IKK2) and a docking/scaffold-like protein NF-κB essential modulator (NEMO, also called IKKγ) that licenses IKKβ activation in response to a host of stress-like stimuli. Activated IKKβ phosphorylates IκBα, targeting it for proteosomal destruction, thereby liberating canonical NF-κB p65/p50 to drive pro-inflammatory gene expression programs {reviewed in [45]–[48]}. In contrast to IKKβ, IKKα functions in the IKK signalosome as the essential kinase that activates the non-canonical NF-κB pathway {reviewed in [45]–[47], [49]}. IKKα-phosphorylated p100 is processed by the proteasome to liberate the NF-κB p52 subunit from its amino terminus, which functions in NF-κB RelB/p52 heterodimers to activate NF-κB target genes regulating adaptive immune responses, cellular survival, and differentiation programs [50], [51]. In addition, IKKα/CHUK functions as a chromatin activating kinase that drives gene transcription in part by modifying nucleosomal histones, interfering with the activities of transcriptional co-repressors, and also by modifying the activities of transcriptional co-activators [52]–[55].

Importantly, IKKα/CHUK also functions as an essential positive effector of terminal keratinocyte differentiation [56]–[58], but independent of both NF-κB and its serine-threonine kinase activity [59]–[61]. During terminal keratinocyte differentiation and cell cycle exit, IKKα controls the transcription of several c-Myc antagonists, including Mad1, Mad2, and Ovol1, by associating with TGFβ-regulated Smad2 and Smad3 heterodimers independent of Smad4 [61]. Due to a complete blockade of terminal keratinocyte differentiation, IKKα KO mice have a perinatal lethal phenotype and a bottle-shaped body morphology with limbs and tails wrapped in thick, sticky epidermal tissue preventing their extension from the body trunk in addition to some skeletal abnormalities likely due to asymmetric ossification effects [56]–[58]. Importantly, IKKα^AA/AA^ knock-in mice (wherein alanines replace Ser^176^ and Ser^180^ T-loop activating phosphorylation sites in IKKα preventing its activation by upstream signaling pathways) are morphologically normal and fertile [62]. Subsequent work showed that abnormal skeletal development in conventional IKKα KO mice was a consequence of failed epidermal differentiation that disengaged normal epidermal-mesodermal interactions and the accumulation of abnormally high levels of specific fibroblast growth factors (FGFs), including FGF-8 and 18 [60]. The latter defective skeletal phenotype is probably due to collateral effects of specific FGFs on BMP (bone morphogenic protein) signaling leading to localized alterations in chondrogenesis or ossification [63]–[65]. Normal skeletal development was restored in *IKKα^−/−^* mice by rescuing IKKα expression solely in the epidermis (*CK14-Ikkα* mice). Because the basal keratinocyte-specific CK14-Ikkα transgene was not expressed in esophageal stratified epithelial tissue, *CK14-Ikkα* mice died 2 days after birth due to a suckling defect stemming from a fused esophagus, [60]. Consequently, the above studies were unable to determine if the IKKα protein, independent of its kinase activity, functionally impacts on the differentiation programs of other cell types as mice progress towards adulthood.

Herein, we have begun to define the mechanisms whereby IKKα positively facilitates articular chondrocyte (AC) ECM remodeling and subsequent chondrocyte differentiation towards hypertrophy. We find that IKKα acts independent of its kinase activity as a mediator of differentiation of both human and murine primary ACs. Our results indicate that IKKα drives chondrocyte differentiation by acting as a positive effector of MMP-13 enzymatic activity via a two pronged mechanism involving the up-regulation of MMP-10 (stromelysin-2) mRNA and the suppression of TIMP-3 accumulation.

## Methods

### Ethics Statement

Research involving human OA patient cartilage tissue samples at the Rizzoli Orthopedic Institute (IOR) was periodically reviewed and approved by the ethics committee/institutional review board of the Institute (“Ethical Committee of IOR), which included documentation of written patient consent forms. Prior to the retrieval of tissues from surgeons, all patient identifiers were removed from tissue samples which were coded by arbitrary number designations to distinguish them solely for experimental purposes.

All work with mice was approved by the IACUC committee of Stony Brook University in accordance with USA NIH guidelines for the use of animals in biomedical research and involved only *in vitro* experiments with knee joint cartilage from 5- to 6-day old mouse pups, which were euthanized by an IACUC approved protocol prior to knee joint dissection.

### Animals

*IKKα^f/f^:CreERT2* homozygous mice containing IKKα alleles flanked by LoxP recombination sites and a 4-hydroxy-tamoxifen (4-OHT) inducible CreERT2 recombinase gene in their Rosa26 loci have been previously described [66]. Exposure of any primary cells isolated from these mice (including primary ACs) to 100 nM 4-OHT {solubilized in 95% ethanol (EtOH)} over a 2 to 3 day time span deletes both IKKα alleles with the subsequent ablation of the endogenous pool of IKKα protein and yields IKKα KO cells.

### Cell Culture

Human primary chondrocytes were isolated from articular cartilage obtained from OA patients undergoing total knee replacement, with approval by IOR ethical committee and written patient consent (as per above Ethics Statement). Chondrocytes were isolated from articular cartilage tissue by sequential enzymatic digestion as previously described [67], and expanded for 1 week in low density monolayer cultures prior to stable transduction with retroviruses or seeding into differentiating pellet cultures as previously described [40], [67]. For experiments to quantify the secretion of specific proteins including activated MMP-13 and activated total collagenases (described in more detail below), conditioned media from pellet cultures were collected with or without overnight serum starvation, centrifuged to eliminate the cellular debris and stored at −20°C for subsequent analysis.

Immature murine articular chondrocytes (iMACs) were isolated from the knee joints of 5- to 6-day-old *IKKα^f/f^:CreERT2* mouse pups as described [68], [69]. After collagenase digestion primary mouse ACs were expanded in DMEM/F12 media supplemented with 10% FBS and antibiotics prior to performing retroviral transductions or inducing their differentiation in high density monolayers or in pellet cultures. For high density monolayer cultures, primary mouse chondrocytes were plated at 2.5×10^4^ cells/cm^2^ in DMEM/F12 supplemented with 10% FBS, antibiotics, 1X ITS Universal Culture Supplement (BDBiosciences), and 50 µg/ml of ascorbic acid (Sigma Aldrich), and kept in confluent cultures for the indicated times with medium change every 48 h. Endogenous IKKα expression was ablated in passage one (P1) IKKα^f/f^:CreERT2 iMACs by 2 to 3 days exposure to 100 nM 4-OHT with EtOH/vehicle treated IKKα^f/f^:CreERT2 iMACs serving as IKKα WT control cells.

### Retroviral Transductions

Knock-down (KD) of IKKα in human OA chondrocytes was achieved by stable transduction of early-passage primary chondrocytes with retroviral vectors expressing a human IKKα-targeted specific shRNA and a puromycin resistance gene, as previously described [40]. Briefly, retroviral transductions were performed by spinoculation with amphotyped viruses prepared with Phoenix A packaging cells; and the resultant IKKα KD chondrocytes were compared with matched wild-type (WT) cells transduced by a puromycin resistance retrovirus harboring a GL2 firefly luciferase–specific shRNA [40].

Rescue of IKKα expression in IKKα KD human OA chondrocytes or in 4-OHT-induced IKKα KO IKKα^f/f^:CreERT2 iMACs was achieved utilizing retroviral vectors co-expressing either neomycin or puromycin resistance, respectively, and a WT or a kinase-dead mutant (K44M) form of murine IKKα, as previously described [70]–[72]. Briefly, viral supernatants in the presence of 8 µg/ml polybrene were applied to cells by centrifugation at ∼1,100 g at 32°C for 45 min. with continued incubation for 5 h at 32°C in 5% CO2 followed by replacement with regular growth medium. At 48 h post-retroviral transduction, primary human OA chondrocytes were selected for neomycin resistance (400 µg/ml) over 12 days, whereas iMACs were selected for puromycin resistance (1.5 µg/ml) over 6 days. Stable retroviral transductions of primary human OA ACs were done with neat (undiluted) viral supernatants prepared with Phoenix A packaging cells [40], while iMACs were infected with retroviral supernatants diluted 3-fold in complete media.

### Microarray Analysis

Total cellular RNAs were isolated from pellet cultures of human OA chondrocytes, as previously described [67], purified by RNeasy Mini spin columns (QIAGEN), labeled following the Affymetrix GeneChip Expression Analysis protocol, and hybridized to Affymetrix HG-U133Plus arrays. Affymetrix CEL files were background-subtracted and normalized by rma (Robust Multichip Average) using a Bioconductor package essentially as previously described [73]. Differential gene expression was evaluated by a modified t test (limma, p val<0.02) and hierarchical clustering was done with Pearson correlation metric and complete linkage analysis also essentially as previously described [70], [71], [73]. Routine quality controls were performed on array data to check for the presence of artifacts and for the consistency of normalization across arrays, showing that results were of good quality and data were comparable.

### Reverse Transcriptase Quantitative PCR (qRT-PCR) Analysis

Total cellular RNA was isolated from pellet cultures of human OA chondrocytes and submitted to qRT-PCR as previously described [40], [67]. PCR primer pairs for the following human genes were used: *GAPDH* (NM_002046, forward 579–598: TGGTATCGTGGAAGGACTCA and reverse 701–683: GCAGGGATGATGTTCTGGA); *TIMP3* (NM_000362.4 forward 1193–1211: CCTTGGCTCGGGCTCATC and reverse 1313–1333: GGATCACGATGTCGGAGTTG) and *MMP10* (NM_002425.2, forward 1278–1298: GCCAGTCCATGGAGCAAGGCT; and reverse 1472–1449: TCGCCTAGCAATGTAACCAGCTGT). Annealing temperatures were 58°C for *GAPDH* and *MMP10* and 60°C for *TIMP3*; mRNA expression levels were normalized to the expression of *GAPDH*, as previously described [21], [40], [74].

For murine chondrocytes total cellular RNAs were isolated from monolayer and pellet cultures using TRIzol® reagent (Life Technologies) followed by DNaseI treatment and column clean-up (QIAGEN); and 250 ng of total RNA were reverse transcribed using the QuantiTect Reverse Transcription Kit (QIAGEN) according to the manufacturer’s instructions. Amplifications were carried out using SYBR Green I-based RT-PCR on the Opticon 2 Real Time PCR Detector System (BioRad), using the following PCR primers: *Col10a1* (NM_009925.4; forward 5′-ACGCATCTCCCAGCACCAGAATC-3′ and reverse 5′-GGGGCTAGCAAGTGGGCCCT-3′); *Runx2* (NM_001145920.2; forward 5′-TCCCCGGGAACCAAGAAGGCA-3′ and reverse 5′-AGGGAGGGCCGTGGGTTCTG-3′); *Mmp10* (NM_019471.2; forward 5′- GCAGCCCATGAACTTGGCCACT-3′ and reverse 5′- AGGGACCGGCTCCATACAGGG-3′); *Mmp13* (NM_008607.2; forward 5′-ATGGTCCAGGCGATGAAGACCCC-3′ and reverse 5′-GTGCAGGCGCCAGAAGAATCTGT-3′); *Vegfa* (NM_001025250.3; forward 5′-CTCGCAGTCCGAGCCGGAGA-3′ and reverse 5′-CAGCCTGGGACCACTTGGC-3′); *Hprt* (NM_013556.2; forward 5′-CAAACTTTGCTTTCCCTGGT-3′ and reverse 5′-CAAGGGCATATCCAACAACA-3′); *Gapdh* (NM_008084.2; forward 5′-GGGCTCATGACCACAGTCCATGC-3′ and reverse 5′-CCTTGCCCACAGCCTTGGCA-3′). Annealing temperatures were 57°C for *Runx2*, *Mmp10* and *Hprt*, and 60°C for *Col10a1*, *Mmp13*, *Vegfa* and *Gapdh*. The data were calculated as the ratio of each gene to *Hprt1* using the 2^−ΔΔCt^ method for relative quantification [75]. *Gapdh* was utilized as an additional housekeeping gene control. Amplification efficiencies were calculated for all primers utilizing serial dilutions of pooled cDNA samples; melting curves were generated to ensure a single gene-specific peak, and no-template controls were included for each run and each set of primers to control for non-specific amplifications.

### Immunoblotting

For detecting TIMP-3 protein, pellet cultures were lysed and total cellular proteins representing the content of ∼80,000 cells were resolved on a 4–12% precast gradient gel (Invitrogen) and transferred to a polyvinylidene difluoride (PVDF) membrane (Millipore). Signals were detected with secondary antibodies and revealed with an ECL Advance kit (Amersham) as previously described [39]. TIMP-3 protein in 1-week pellet cultures was detected using a mouse monoclonal anti-human TIMP-3 antibody (R&D Systems). To detect exogenous IKKα proteins introduced by retroviral transduction, whole cell lysates from monolayer cultures were evaluated by Western blotting on membranes that were probed initially with rabbit polyclonal anti-IKKα antibody (CST Inc.) and subsequently stripped and re-probed with a rabbit polyclonal anti-hemaglutinin epitope (HA) antibody (Zymed) to specifically detect HA-tagged murine WT IKKα and mutant IKKα(K44M) proteins. All blots were re-probed with a rabbit polyclonal anti-β-tubulin antibody (SIGMA) as protein loading control.

Immunoblotting of proteins in mouse chondrocytes was performed with whole cell lysates of monolayer cultures, transferred to PVDF membranes and incubated with primary antibodies against IKKα (CST Inc.), IKKβ (CST Inc.), HA (Zymed), or MMP-10 (Novus Biological). β-tubulin (Abcam) was used as loading control. Relative abundance of MMP-10 protein was determined by densitometry, using ImageJ software (NIH, Bethesda, MD), and normalized to β-tubulin.

### Immunohistochemistry and Immunocytochemistry

Frozen sections (5 µm) of 1-week pellet cultures of human OA chondrocytes were analyzed by immunohistochemistry (IHC) in conjunction with hematoxylin nuclear counterstaining (CAT hematoxylin – BIOCARE Medical) using antibodies against type II collagen (COL2), Runx2, type×collagen (COL10) and the antibody C1,2C (IBEX Inc.) against a specific COL2–3/4C neo-epitope, as previously described [40]. TIMP-1, TIMP-2, TIMP-3 and TIMP-4 proteins were detected with mouse monoclonal antibodies (R&D Systems). MMP-10 protein was detected by a rabbit polyclonal anti-human/mouse antibody (Novus Biological). Differentiating micromasses were analyzed for the presence and relative abundance of the above proteins by *in situ* antibody staining followed by quantitative imaging analysis, as we had previously described [39], [40]. Briefly, this quantitative, *in situ* technique involved the analysis of 4–5 400X magnified fields obtained in 2 sequential sections from each micromass sphere, which were processed and quantified with a Nikon Eclipse 90i microscope equipped with Nikon Imaging Software elements. Relative protein levels were set to a threshold level based on isotype control staining; and the data were expressed as the percentage of signals per µm^2^ unit area. Control experiments verified that quantified results obtained with 2 sequential micromass sections were comparable to data obtained with sections from different depths within each micromass sphere (data not shown).

*In situ* levels of proteins were also visualized in 1- and 3-week pellet and high density monolayer cultures of mouse primary chondrocytes. Briefly, OCT-embedded pellets were sectioned into 5 µm slices and incubated with specific antibodies against COL2 (Santa Cruz Biotechnology, Inc.), followed by incubation with an anti-goat horseradish peroxidase (HRP) conjugated secondary antibody (Santa Cruz Biotechnology, Inc.); or rabbit polyclonal antibodies against COL10 (Abcam), MMP-10 (Novus Biological) or C1,2C (IBEX) followed by incubation with an anti-rabbit Alexa Fluor® 555 conjugated secondary antibody (CST Inc.). Immunocytochemistry was performed in high-density monolayer cultures of mouse primary chondrocytes seeded in culture-treated coverslips (NUNC). At 3 weeks after plating, cells were fixed and incubated with antibodies against COL2 (Santa Cruz Biotechnology, Inc.) or COL10 (Abcam) followed by incubation with anti-goat Alexa Fluor 488 conjugated (Life Technologies) or anti-rabbit Alexa Fluor® 555 conjugated antibodies (CST Inc.), respectively. Sections were mounted with anti-fade mounting medium containing DAPI (Vector Labs). Immunostainings were visualized with a fluorescence microscope (NIKON ECLIPSE E800) and images were taken (SPOT RT Diagnostic).

### MMP-10, MMP-13 and Total Collagenase Assays

MMP-10 protein levels present in the conditioned media of 1-week pellet cultures of human OA chondrocytes were quantified by the Quantikine® Human Pro-MMP-10 ELISA-based assay (R&D Systems), which employs a quantitative sandwich enzyme immunoassay technique to evaluate MMP-10 pro-enzyme levels. Standards and undiluted culture medium were incubated for 2 h at room temperature in a microplate pre-coated with a monoclonal anti-human MMP-10 antibody; after washing, a monoclonal HRP-conjugated anti-human MMP-10 antibody was added to the wells. After washing to remove unbound antibody, substrate solution (tetramethylbenzidine+hydrogen peroxide) was added to the wells with color developing in proportion to the amount of pro-MMP-10. Optical densities at 450 nm were recorded within 30 min with a microplate reader (ELISA reader Multiskan HCC340_Labsystems) and protein quantities in pg/ml were determined on a standard curve. Sample diluent background signals were subtracted from each value. The standard curve was generated by reducing the data with a software package that provides four parametric logistic curve fit (Genesis Lite 3.03, Life Sciences (UK) Ltd.).

MMP-13 activity in the conditioned medium of 1- to 3-week human OA chondrocyte pellet cultures was quantified with a SensoLyte® Plus 520 MMP-13 Assay Kit (AnaSpec). A monoclonal anti-human-MMP-13 antibody was coated on microplates to pull down both pro and active forms of MMP-13 from undiluted culture medium. Standards and undiluted medium were incubated for 2 h at room temperature in pre-coated microplates. Activated levels of MMP-13 were detected with a 5-FAM/QXL^TM^520 FRET peptide substrate. The fluorescence of 5-FAM (fluorophore) was quenched by QXL™520 (quencher) in the intact FRET peptide. Upon MMP-13 cleavage, the fluorescence of 5-FAM was recovered and monitored at Ex/Em = 490±20 nm/520±20 nm. Substrate was added, and after 1 hour incubation, the fluorescence values were taken with a microplate reader (SpectraMax Gemini_Molecular Devices). The MMP-13 standard curve was generated by incubating serial dilutions of recombinant pro-MMP-13 in a microtiter plate together with 1 mM APMA (4-amino­phenyl­mercuric acetate) activator; and a four parametric logistic curve-fit was obtained with Genesis Lite 3.03 software to convert relative fluorescence units (RFU) to ng/ml of MMP-13 enzyme. Background fluorescence readings in enzyme minus control reactions were subtracted from each value.

Total collagenase activity in the conditioned media of either human or murine chondrocytes cultures was quantified with an EnzChek®Gelatinase/Collagenase Assay Kit (Molecular Probes). The assay employs a DQ™ type I collagen fluorescein conjugate substrate that releases fluorescent peptides after collagenase cleavage with increases in fluorescence being proportional to the levels of proteolytic activity. Standards and undiluted culture media were incubated with DQ™ type I collagen and fluorescence readings were taken after 2.5 hours (human) or 6 hours (mouse) with a microplate reader (SpectraMax Gemini_Molecular Devices) at Ex/Em = 490/530 cutoff 515. A standard curve to convert RFU to Units/ml was generated by serial dilution of 1 mM APMA activated Clostridium Collagense Type IV. Background fluorescence readings in enzyme minus control reactions were subtracted from each value.

### Alizarin Red Staining

Calcium-containing mineral deposits were visualized and quantified by Alizarin red staining (Sigma Aldrich) in high density iMAC monolayer cultures differentiated for the indicated times. Briefly, cells were fixed in 10% formaldehyde for 15 min and exposed to a 2% solution of Alizarin red pH 4.2 for 60 min at room temperature. Stained cells were extensively washed with PBS to remove the nonspecific precipitation and matrix mineralization was quantified by extraction of the Alizarin Red S dye. To do so, cells were lysed in PBS containing 0.01% Triton X-100; lysates were cleared by centrifugation at 10,000×g for 5 min. at room temperature; and dye-containing supernatants were extracted and aliquoted into 96 well plates for quantification at 492 nm with a plate reader. All measurements were performed in triplicate.

### Statistical Analysis

All data are expressed as the mean ± S.E.M (error bars). Means of groups were compared with GraphPad Prism 5.00 statistical software (GraphPad Prism Software, Inc.) by one of several different statistical tests including Wilcoxon matched pairs test, ANOVA or Student’s t test, with the choice of statistical test depending on unique details or aspects of each experiment, as indicated in the Figure Legends.

## Results

### IKKα in Articular Chondrocytes Functions as a Facilitator of ECM Remodeling and Subsequent Steps in Differentiation Towards Hypertrophy

**Figure 1:**
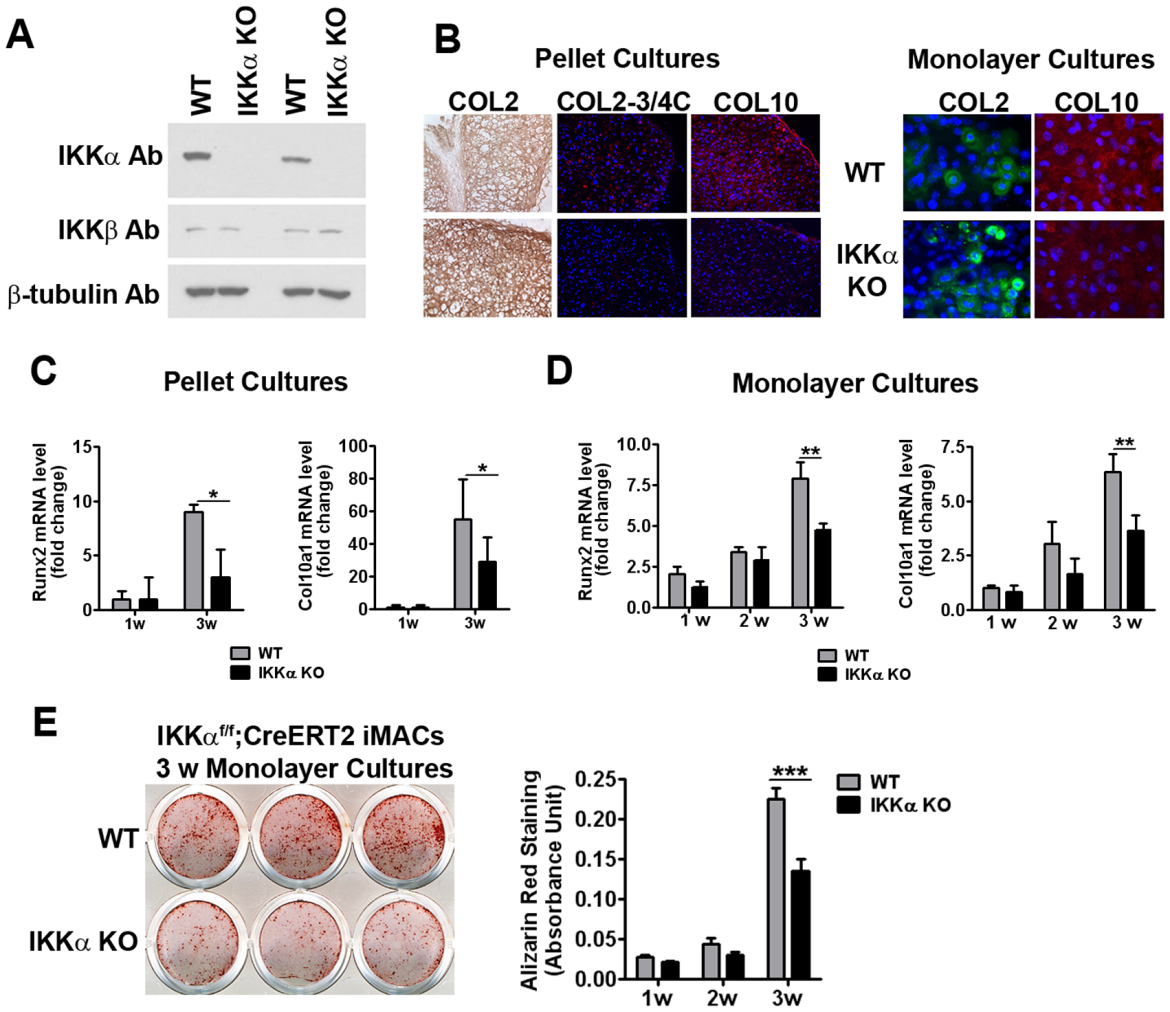
Effects of IKKα ablation on iMAC differentiation. (**A**) IKKα immunoblot of total cell lysates from two independent experiments using IKKα^f/f^;CreERT2 immature articular chondrocytes (iMACs) treated with EtOH vehicle (WT control) or 4-OHT (IKKα-KO) for 72 hours. IKKβ and β-tubulin were used as 4-OHT specificity and protein loading controls, respectively. (**B**) (Left): Relative levels of IHC staining with COL2, C1,2C and COL10 antibodies in 3-week pellet cultures of IKKα^f/f^;CreERT2 iMACs treated with EtOH vehicle (WT control) or 4-OHT (IKKα KO). Results are representative of 3 experiments. (Right): COL2 and COL10 immunopositive cells in 4-OHT (IKKα KO) treated IKKα^f/f^;CreERT2 iMAC 3-week monolayer cultures vs. their matched WT (EtOH treated) controls. Results are representative of 2 (COL2) and 3 (COL10) independent experiments (Original magnification, 200X). Merged immunofluorescence images are shown. COL2 (monolayer cultures): green; COL10 (pellet and monolayer cultures): red; COL2–3/4C (pellet cultures): red; DAPI: blue. See Figure S1 for comparable results of other experiments. (**C**) qRT-PCR analysis of Runx2 (left) and Col10a1 (right) RNA in EtOH (WT) or 4-OHT (IKKα KO) treated IKKα^f/f^;CreERT2 iMAC 1- and 3-week pellet cultures. Data are displayed as fold-change of 3-week vs. 1-week cultures, and shown as mean ± S.E.M. of 3 experiments. *p<0.05 by Student’s t-test. (**D**) qRT-PCR analysis of Runx2 and Col10a1 RNAs in IKKα^f/f^;CreERT2 iMAC monolayer cultures treated with EtOH (WT) or 4-OHT (IKKα KO). Data are shown as fold change relative to 72 hour EtOH (WT) and 4-OHT (IKKα KO) treated cultures of at least 4 experiments. **p<0.01 by ANOVA. (**E**) (Left): Representative Alizarin red staining of EtOH (WT) and 4-OHT (IKKα KO) treated 3-week IKKα^f/f^;CreERT2 iMAC high density monolayer cultures. (Right): Quantification of Alizarin red staining after dye solubilization of EtOH (WT) and 4-OHT (IKKα KO) monolayer cultures differentiated as indicated. Data are mean ± S.E.M. of 3 experiments, each performed in duplicate. ***p<0.001 by ANOVA.

To investigate the general significance of an IKKα requirement to drive the *in vitro* differentiation of ACs, which we had previously reported for primary human OA derived chondrocytes [40], we compared the *in vitro* ECM and differentiation status of wild type (WT) and 4-OHT-induced IKKα knockout (KO) chondrocytes isolated from *IKKα^f/f^;CreERT2* 5- to 6 day-old mice [66]. As shown in Figure 1A, three days of 4-OHT treatment specifically ablated the expression of IKKα protein in IKKα^f/f^;CreERT2 iMACs. Initially, we assessed the relative ECM remodeling capabilities of differentiating pellet cultures of primary WT (EtOH vehicle-treated) and IKKα KO (4-OHT treated) iMACs by IHC analysis with antibodies against specific ECM components (Figure 1B, Left). We observed enhanced COL2 deposition in 4-OHT-induced IKKα KO iMACs in comparison to matched WT controls (Figure 1B, Left and Figure S1). Further, we detected decreased MMP-generated COL2–3/4C neo-epitopes and decreased COL10 protein (a well-accepted marker of hypertrophic chondrocytes) [10], [12], [76] in the IKKα KO cells compared to WT controls (Figure 1B, Left and Figure S1). For additional verification of these observations, similar experiments were done with independent cultures of differentiating 3-week high-density monolayer iMACs, which produced comparable results (Figure 1B, right and Figure S1). Next, we determined the impact of IKKα ablation on Col10a1 and Runx2 mRNA levels in both iMAC pellet and high-density monolayer cultures (Figure 1C and 1D, respectively). In both cases, the absence of IKKα in 3-week differentiating chondrocytes significantly reduced the expression of both Col10a1 and Runx2 mRNAs (Figures 1C and 1D). To determine if the loss of Col10a1 and Runx2 expression in IKKα KO iMACs was indicative of a clear deficiency in terminal differentiation capacity, we evaluated the same cells for calcium deposition, a defining feature of chondrocyte hypertrophy. Indeed, Alizarin red staining revealed significantly enhanced calcium deposition in WT control iMACs vs. their matched IKKα KO counterparts (Figure 1E).

### IKKα is Required to maintain MMP-10 (stromelysin-2) Levels in Differentiating Articular Chondrocytes

**Figure 2:**
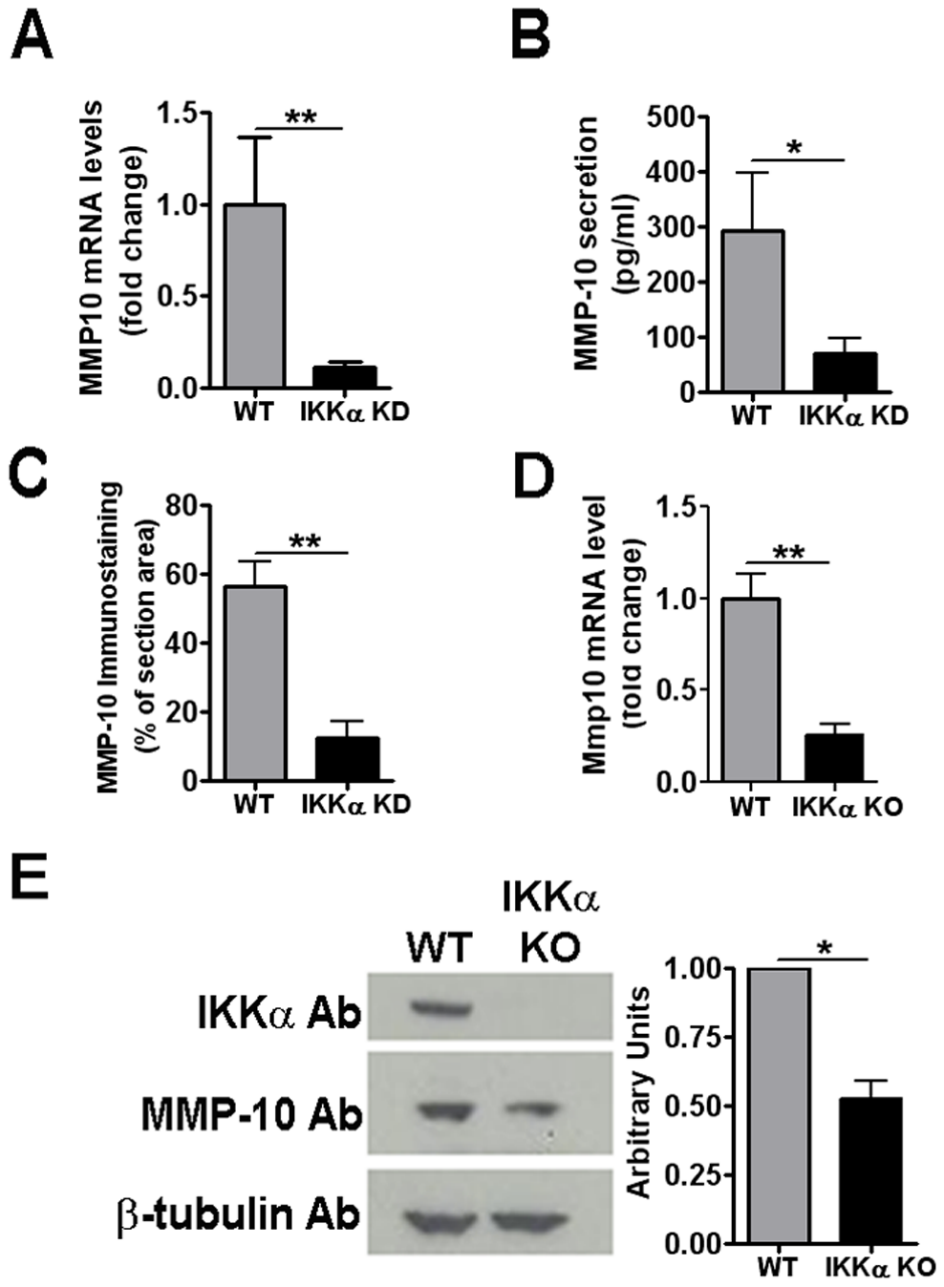
Effects of IKKα loss on MMP-10 levels in differentiating human and murine chondrocytes. (**A**) qRT-PCR analysis of MMP10 RNA in 1-week differentiated pellet cultures of IKKα KD vs. WT (GL2 control) human OA chondrocytes. Results were obtained with primary chondrocytes derived from 8 OA patients. Data are shown as mean ± S.E.M. (error bars) and represented as fold-change vs. WT (set as 1.0). **p<0.01 by Wilcoxon matched pair test. (**B**)**:** MMP-10 protein secretion quantified by immunoassay of conditioned medium (performed as described in Methods) of 1-week differentiated pellet cultures (N = 5). Data are shown as mean ± S.E.M. *p<0.05 by Wilcoxon matched pair test. (**C**) MMP-10 immunostaining in 1-week differentiated pellet cultures of IKKα KD vs. WT (GL2 control) human OA chondrocytes (N = 3). Immunostaining expressed as percentage of signal per µm^2^ unit area. **p<0.01 by Student’s t test. Representative images of MMP-10 immunostained micromasses are presented in Figure 3. (**D**) qRT-PCR analysis of Mmp10 RNA in 4-OHT (IKKα KO) or EtOH vehicle-treated (WT) 1-week high density chondrocyte monolayer cultures of IKKα^f/f^;CreERT2 iMACs. Data are represented as fold-change relative to the WT control (set as 1.0) and shown as mean ± S.E.M. of 3 independent experiments. **p<0.01 by Student’s t-test. (**E**) (Left): Representative MMP-10 immunoblot of whole cell lysates of 1-week EtOH (WT) and 4-OHT (IKKα KO) treated IKKα^f/f^;CreERT2 high density iMAC monolayer cultures. (Right): Densitometric analysis of MMP-10 immunoblots shown as mean ± S.E.M. of 3 independent experiments. *p<0.05 by Student’s t test.

To begin to explore mechanisms of action of IKKα as a positive effector of chondrocyte ECM remodeling and subsequent differentiation, we employed whole genome RNA expression profiling as a primary screen to identify mRNA targets of IKKα in differentiating primary human OA chondrocytes. Gene expression profiling was performed with HgU133plus Affymetrix arrays and total cellular RNA preparations isolated from IKKα KD and matched WT 1-week pellet cultures, which were prepared with primary ACs derived from 3 OA patients. As shown in Figure S2, hierarchical clustering representation of differentially expressed genes revealed that IKKα KD cells were profoundly deficient in the expression of the gene encoding MMP-10 (stromelysin 2) [37]. We validated these microarray results by performing qRT-PCR analysis on total cellular RNAs isolated from 1-week pellet cultures prepared with primary ACs derived from 8 OA patients. In agreement with our array data, qRT-PCR analysis revealed strong repression of MMP10 mRNA in IKKα KD chondrocytes (see Figure 2A).

**Figure 3:**
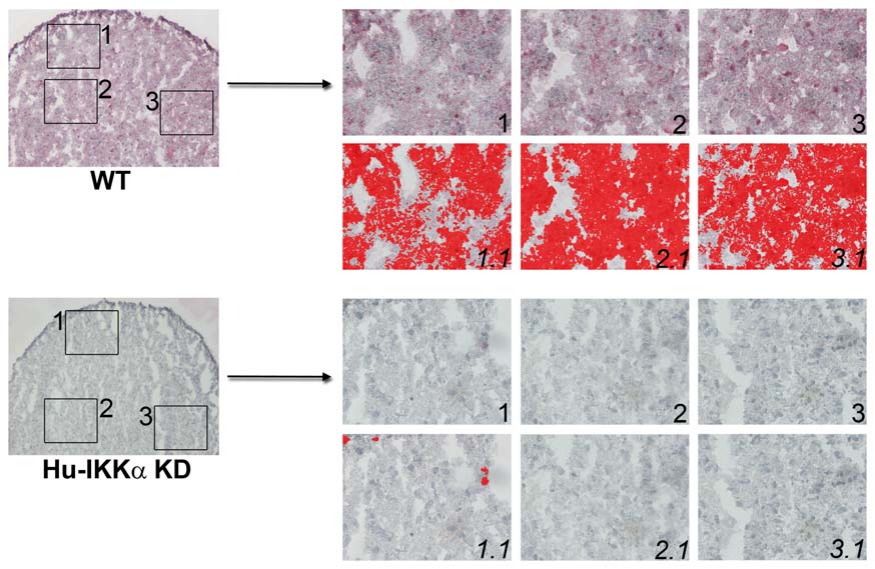
Effects of IKKα ablation on MMP-10 protein levels in human OA chondrocyte micromass cultures. MMP-10 immunostaining in 1-week differentiated pellet cultures of IKKα KD vs. matched WT (GL2 control) human OA chondrocytes. Global views of micromass sphere sections (100Xmagnification) are shown on the left; and three random fields at 400Xmagnification, which were submitted to quantitative image analysis appear to the right. Antibody stained images are shown in the right side upper rows (labeled as 1, 2 & 3), with each 400Xfield submitted to analysis by Nikon Imaging Software in the lower rows (1.1, 2.1 & 3.1). For each sample an IHC staining threshold was established based on an antibody isotype control. Antibody staining of MMP-10 is in red in conjunction with hematoxylin (blue) nuclear counterstaining. Data are representative examples of multiple sections of micromasses prepared with ACs of 3 OA patients; and results of all such experiments are presented as statistically analyzed bar graphs in Figure 2C.

Next, we used a quantitative ELISA assay to determine the effect of IKKα KD on MMP-10 protein levels present in the conditioned media of an independent set of 1-week pellet cultures derived from 5 OA patients, which showed a comparable pronounced reduction in secreted MMP-10 protein (Figure 2B). These results were independently confirmed by *in situ* IHC staining and quantitative imaging analysis of MMP-10 protein levels in frozen sections of IKKα KD and WT (GL2 control) human OA chondrocyte pellet cultures (Figures 2C and 3).

In parallel with the above experiments, we determined if MMP10 was also a target of IKKα in inducible IKKα KO iMACs. Indeed, 4-OHT-treated IKKα^f/f^;CreERT2 chondrocytes displayed a very significant reduction in Mmp10 mRNA compared to vehicle-treated WT controls in high density monolayer cultures (Figure 2D) and also in 1-week pellet cultures (data not shown). MMP-10 immunoblotting performed with total cellular proteins isolated from independent high density iMAC monolayer cultures also showed significantly decreased MMP-10 protein levels in the absence of IKKα (Figure 2E).

### IKKα Post-transcriptionally Regulates TIMP-3 Protein Levels

**Figure 4:**
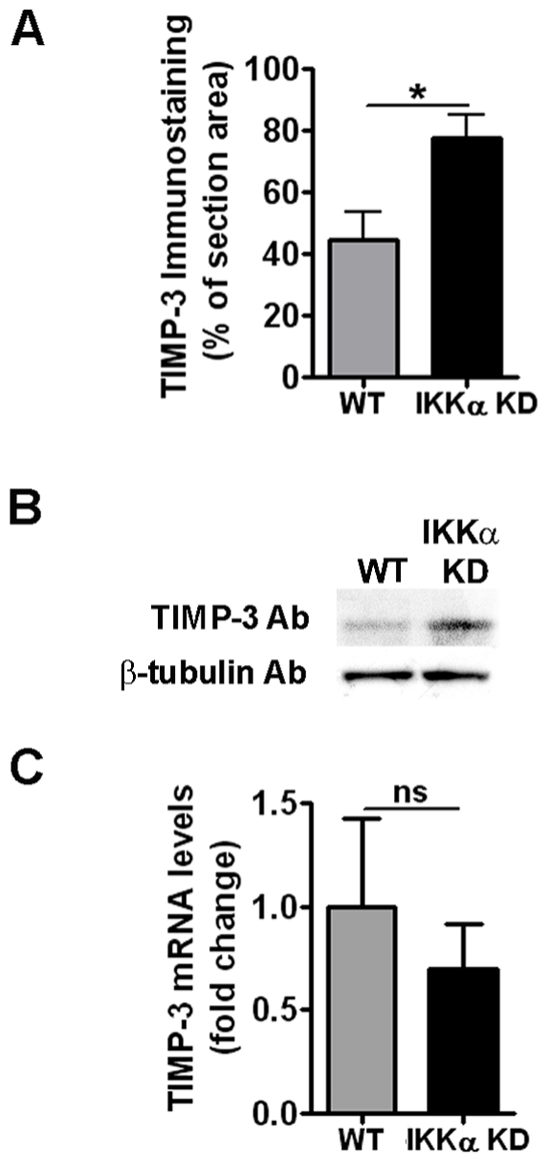
Effects of IKKα KD on TIMP-3 levels in human OA chondrocyte micromass cultures. (**A**) Quantitative image analysis for TIMP-3 immunostaining of 1-week IKKα KD human pellet cultures vs. matched WT (GL2 control) for 3 OA patients. Results are shown as mean ± S.E.M. *p<0.05 by Student’s t-test. Representative images of TIMP-3 immunostained micromasses are presented in Figure 5. (**B**) TIMP-3 immunoblot of whole cell lysates of IKKα KD and matched WT (GL2 control) of 1-week human chondrocyte pellet cultures of one representative OA patient, with β-tubulin as protein loading control. (**C**) qRT-PCR analysis of TIMP3 mRNA in 1-week pellet cultures of IKKαKD human chondrocytes vs. matched WT (GL2) controls (N = 6). Results are shown as mean ± S.E.M. ns = not significant.

**Figure 5:**
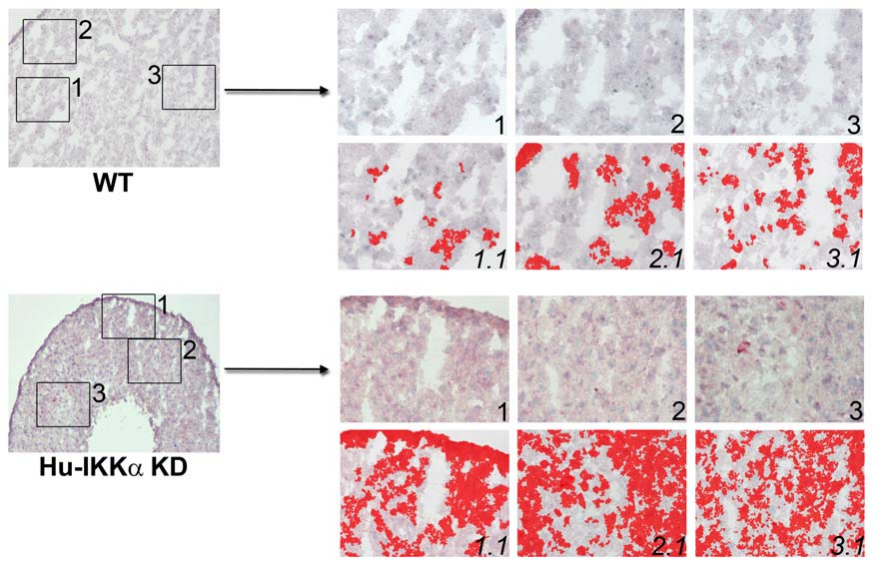
IKKα KD increases TIMP-3 protein levels in human OA chondrocyte micromass cultures. TIMP-3 immunostaining in 1-week differentiated pellet cultures of IKKα KD vs. matched WT (GL2 control) human OA chondrocytes. Global views of micromass sphere sections (100Xmagnification) are shown on the left; and three random fields at 400Xmagnification, which were submitted to quantitative image analysis, appear to the right. Antibody stained images are shown in the right side upper rows (labeled as 1, 2 & 3), with each 400Xfield submitted to analysis by Nikon Imaging Software in the lower rows (1.1, 2.1 & 3.1). For each sample an IHC staining threshold was established based on an antibody isotype control. Antibody staining of TIMP-3 is in red in conjunction with hematoxylin (blue) nuclear counterstaining. Data are representative examples of multiple sections of micromasses prepared with ACs of 3 OA patients; and results of all such experiments are presented as statistically analyzed bar graphs in Figure 4A.

To determine if the mechanism of action of IKKα as a regulator of ECM remodeling involved other targets besides the pro-collagenase activator MMP-10, we next investigated if any of the tissue inhibitors of MMPs (TIMPs) were also subject to regulation by IKKα. Among the four major TIMPs, only TIMP-3 inhibits both MMPs and aggrecanases in chondrocytes [38]. Interestingly, quantitative IHC imaging analysis revealed significant enhancement of TIMP-3 protein levels in the ECM of 1-week IKKα KD pellet cultures in comparison to their matched WT controls (Figures 4A and 5), which was independently confirmed in immunoblots of whole cell lysates (Figure 4B). However, there was no significant change in TIMP3 mRNA levels in IKKα KD samples generated with ACs from 3 OA patients (Figure 4C). Similar experiments performed to assess the levels of the other TIMP proteins (TIMP-1, 2 and 4) failed to show evidence of significant, IKKα-dependent effects in the same 1-week pellet cultures (data not shown).

### IKKα Positively Modulates Total Collagenase and MMP-13 Activities in Differentiating Chondrocytes

**Figure 6:**
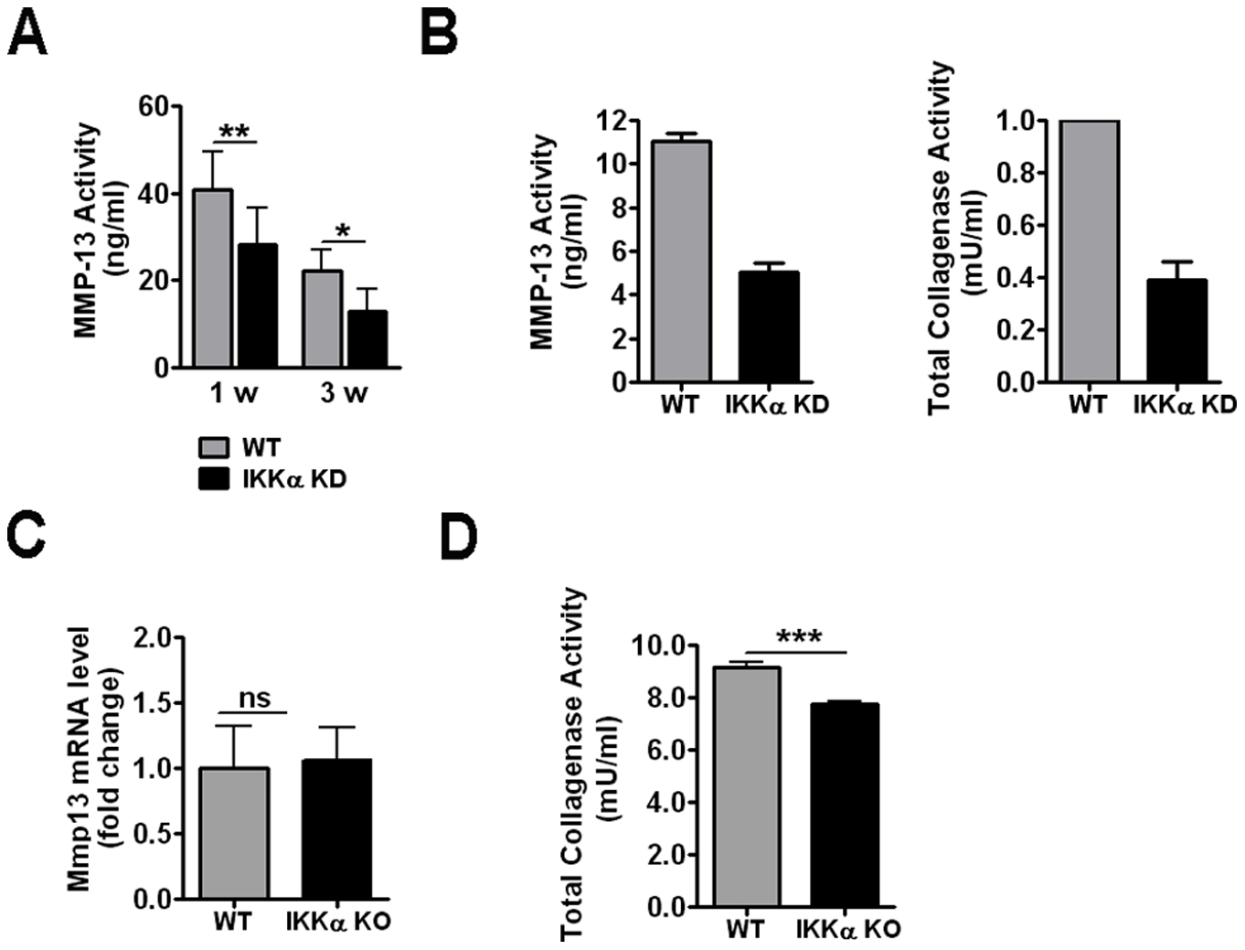
Effects of IKKα loss on MMP-13 and total collagenase activities. (**A**) MMP-13 activity in conditioned medium of 1-week (N = 9) and 3-week (N = 7) IKKα KD pellet cultures of human OA chondrocytes. Data are represented as mean ± S.E.M. (error bars). *p<0.05, **p<0.01 by Wilcoxon matched pair test. (**B**) MMP-13 (Left) and total collagenase (Right) activities in serum-free conditioned media of 1-week IKKα KD and matched WT (GL2 control) pellet cultures of ACs from 2 OA patients. Data are represented as mean ± S.E.M. (error bars). (**C**) qRT-PCR analysis of Mmp13 expression in 1-week high density monolayer cultures of IKKα^f/f^;CreERT2 iMACs treated with EtOH (WT control) or 4-OHT (IKKα KO). Data are from 3 independent experiments and shown as mean ± S.E.M. (error bars). ns = not significant by Student’s t test. (**D**) Total collagenase activity quantified in the conditioned medium of 1-week EtOH (WT control) and 4-OHT (IKKα KO) treated IKKα^f/f^;CreERT2 iMACs. ***p<0.001 by Student’s t test.

In light of the effects of IKKα loss on MMP-10 and TIMP-3 levels, we next performed quantitative assays to directly evaluate the effects of IKKα loss on MMP-13 and total collagenase activities. To this end, we utilized conditioned medium of 1- and 3-week IKKα KD and WT control pellet cultures established with ACs obtained from multiple OA patients (Figure 6A and B). We observed statistically significant decreases in MMP-13 activity of between 30 and 40% in the IKKα-deficient cells compared to their matched WT controls at both time points (Figure 6A). To investigate if serum in the cell growth media affected these results, we also evaluated MMP-13 activity as a function of IKKα in the serum-deprived conditioned media from 1-week pellet cultures prepared with the ACs of 2 OA patients and observed ∼2-fold diminutions in both MMP-13 and total collagenase activities under these alternative assay conditions (Figure 6B). In agreement, independent experiments performed with iMACs showed that, even though IKKα ablation did not affect Mmp13 expression levels (Figure 6C), the total collagenase activity was decreased in IKKα KO chondrocytes and therefore dependent on IKKα (Figure 6D). Moreover, analogous to our MMP-13 protein and activity data in IKKα KD human OA chondrocytes, IKKα KO iMACs had WT levels of MMP-13 protein but were deficient in MMP-13 enzymatic activity (data not shown).

### IKKα Serine-threonine Kinase Activity is not Required for its Effects on ECM Remodeling and Chondrocyte Differentiation

**Figure 7:**
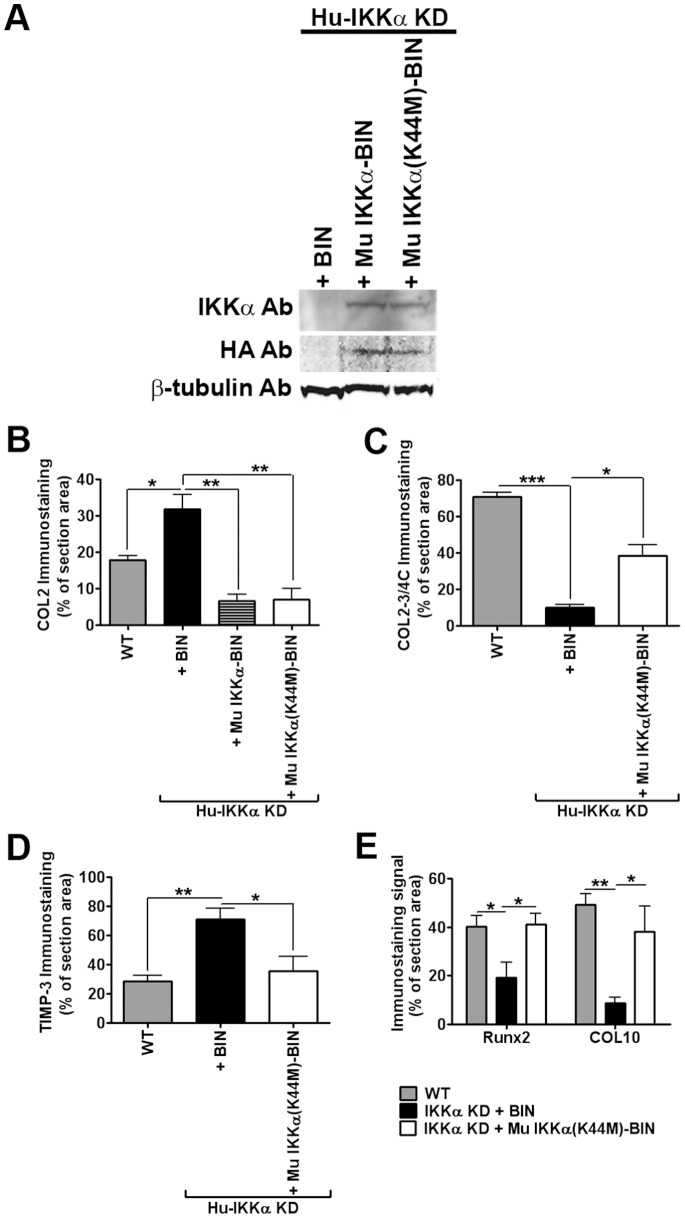
Effects of IKKα on human chondrocyte ECM remodeling and differentiation are independent of its kinase activity. (**A**) Immunoblots of whole cell lysates of monolayer cultures of primary human (Hu) IKKα KD chondrocytes, which were stably transduced with a neomycin-resistant retroviral vector (BIN), or with BIN retroviral vectors co-expressing WT murine IKKα (Mu IKKα-BIN) or a kinase-dead murine IKKα mutant {Mu IKKα(K444M-BIN)}. Blots were first probed with an anti-IKKα antibody and then sequentially re-probed with anti-HA and β-tubulin antibodies. (**B**) Quantitative image analysis of COL2 immunostaining in 1-week pellet cultures of primary human chondrocytes of 3 OA patients: WT (GL2 control), IKKα KD cells with BIN, IKKα KD cells with Mu IKKα-BIN or IKKα KD cells with Mu IKKα(K44M)-BIN. Data are shown as mean ± S.E.M (error bars). *p<0.05 and **p<0.01 by Student’s t-test. (**C**) Quantitative image analysis of C1,2C immunostaining in the same WT (GL2 control), Hu-IKKα KD+BIN and Hu-IKKα KD+Mu IKKα(K44M)-BIN samples as in panel B. Data are shown as mean ± S.E.M. (error bars). *p<0.05 and ***p<0.001 by Student’s t-test. (**D**) Quantitative image analysis of TIMP-3 immunostaining of WT (GL2 control), Hu-IKKα KD+BIN and Hu-IKKα KD+Mu IKKα(K44M)-BIN of 3-week pellet cultures prepared with primary ACs derived from 3 OA patients. *p<0.05 and **p<0.01 by Student’s t test. (**E**) Quantitative image analysis of Runx2 and COL10 immunostaining of the same WT (GL2 control), Hu-IKKα KD+BIN and Hu-IKKα KD+Mu IKKα(K44M)-BIN samples in panels B and C. Data are shown as mean ± S.E.M. (error bars). *p<0.05 and **p<0.01 by Student’s t-test. Representative examples of COL2, COL2–3/4, TIMP-3, Runx2 and COL10 immunostained micromasses, which were submitted to quantitative imaging analysis, are respectively shown in Figures 8–12.

**Figure 8:**
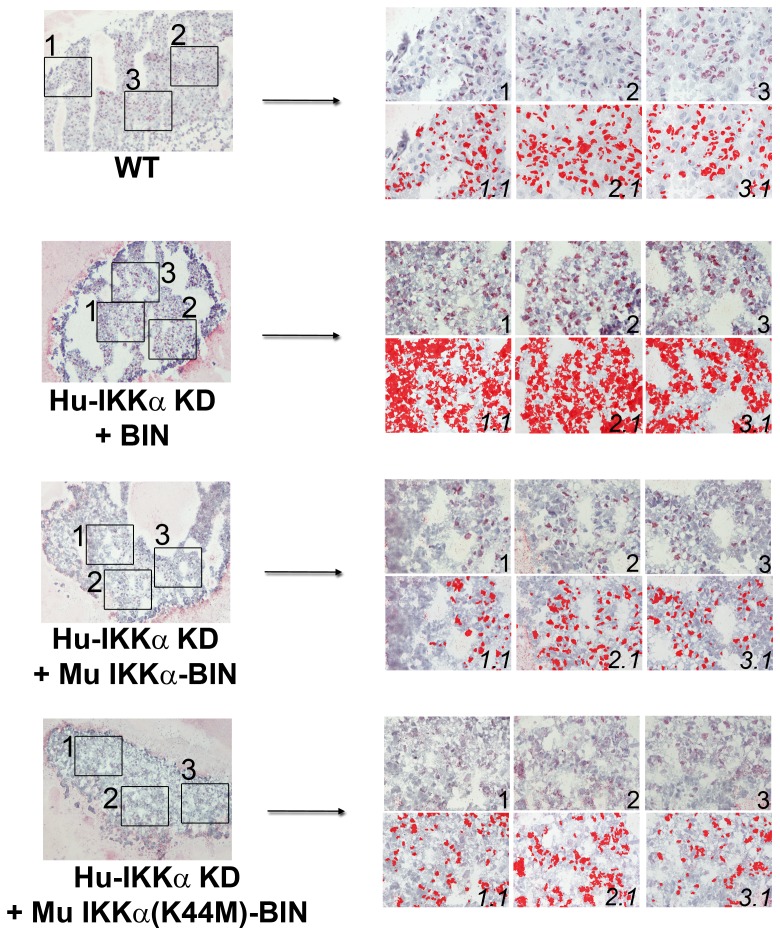
Effects of IKKα on COL2 protein levels in human OA chondrocyte micromass cultures are independent of its kinase activity. COL2 immunostaining in 1-week pellet cultures of primary human OA chondrocytes: WT (GL2 control), IKKα KD cells with empty BIN retroviral vector, IKKα KD cells with WT murine IKKα-BIN and IKKα KD cells with kinase-dead murine IKKα(K44M)-BIN. Global views of micromass sphere sections (100Xmagnification) are shown on the left; and three random fields at 400Xmagnification submitted to quantitative image analysis are on the right. Antibody stained images are shown in the right side upper rows (labeled 1, 2 & 3), with each 400Xfield analyzed by Nikon Imaging Software in the lower rows (labeled 1.1, 2.1 & 3.1). For each sample an IHC staining threshold was established based on an antibody isotype control. Antibody staining is in red in conjunction with hematoxylin (blue) nuclear counterstaining. Data are representative examples of multiple sections of micromasses prepared with ACs of 3 OA patients; and results of all such experiments are presented as statistically analyzed bar graphs in Figure 7B.

**Figure 9:**
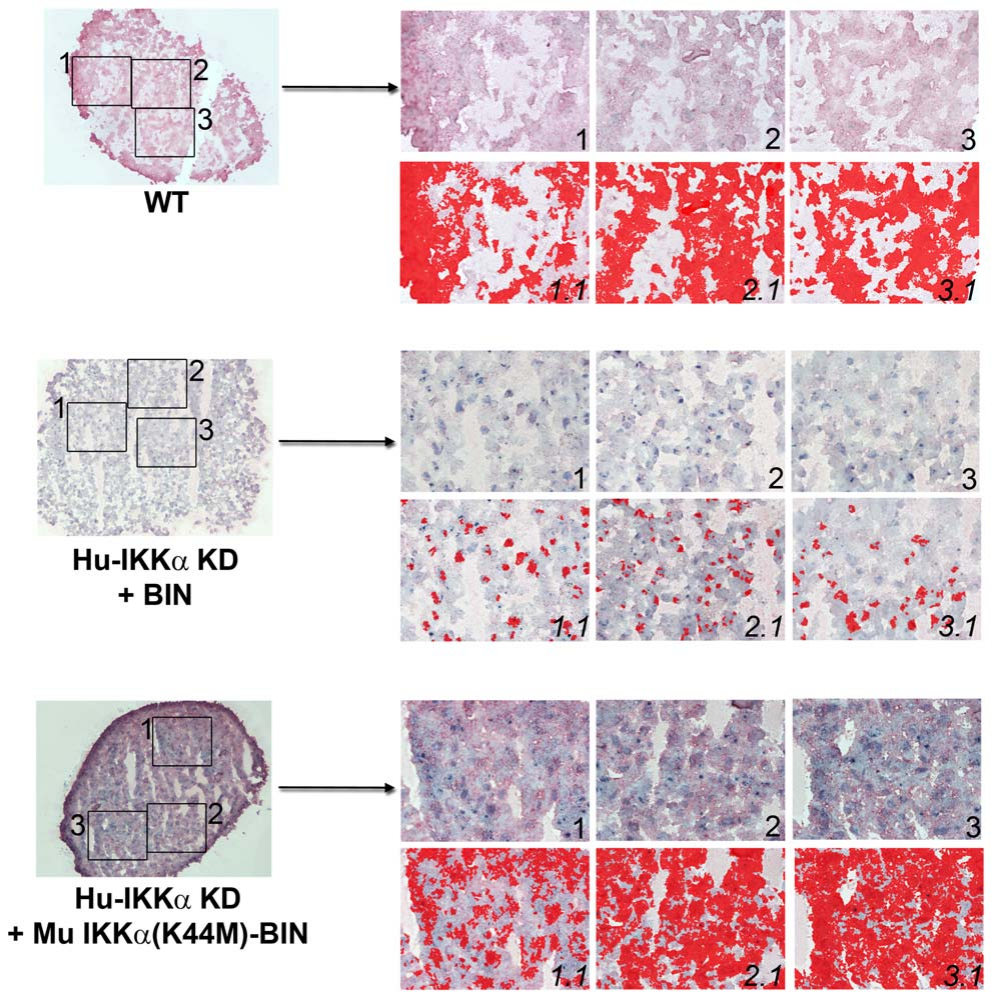
Effects of IKKα on the accumulation of COL2–3/4C neoepitopes in human OA chondrocyte micromass cultures are independent of its kinase activity. C1,2C antibody detection of COL2–3/4C neoepitopes in the same 1-week pellet cultures of primary human chondrocytes employed above in Figure 8. Global views of micromass sphere sections (100Xmagnification) are shown on the left; and three random fields at 400Xmagnification submitted to quantitative image analysis are on the right. Antibody stained images are shown in the right side upper rows (labeled 1, 2 & 3), with each 400Xfield analyzed by Nikon Imaging Software in the lower rows (labeled 1.1, 2.1 & 3.1). For each sample an IHC staining threshold was established based on an antibody isotype control. Antibody staining is in red in conjunction with hematoxylin (blue) nuclear counterstaining. Data are representative examples of multiple sections of micromasses prepared with ACs of 3 OA patients; and results of all such experiments are presented as statistically analyzed bar graphs in Figure 7C.

**Figure 10:**
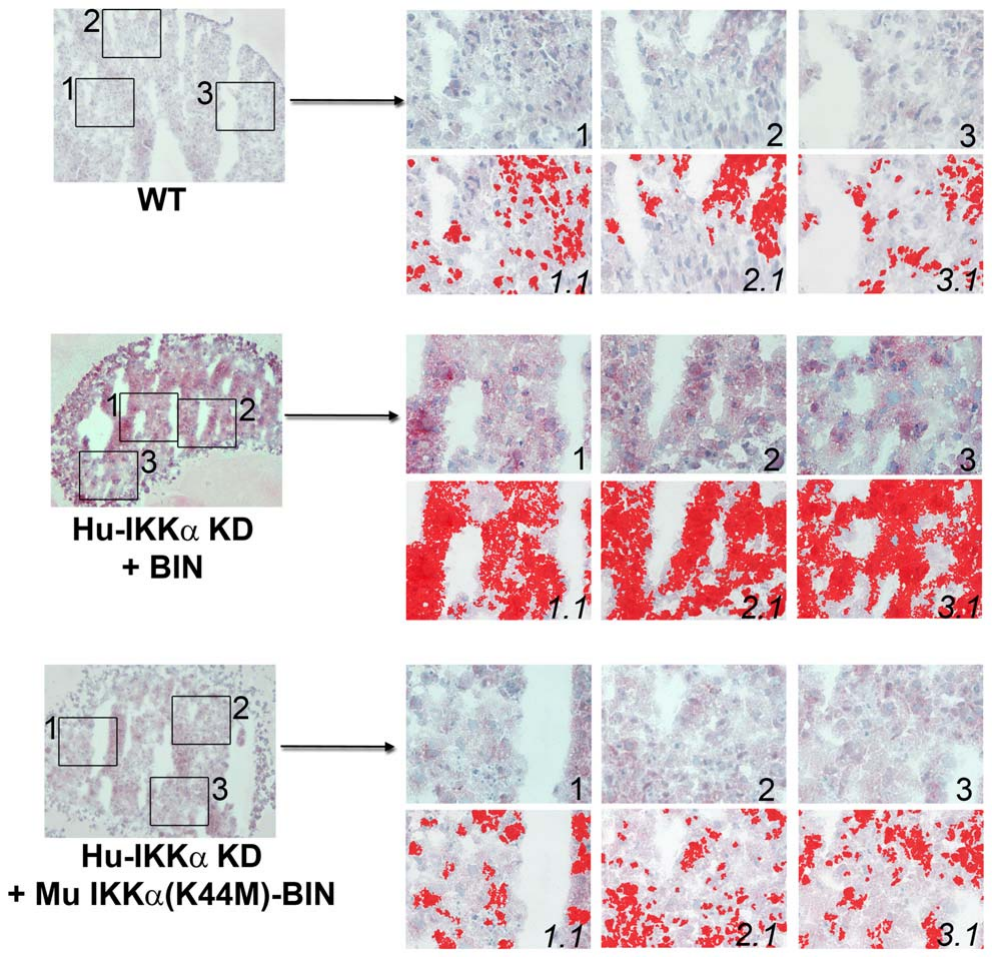
Effects of IKKα knock-down on TIMP-3 levels in human OA chondrocyte micromass cultures are independent of its kinase activity. TIMP-3 immunostaining in 1-week pellet cultures of primary human OA chondrocytes: WT (GL2 control), IKKα KD cells with empty BIN retrovector and IKKα KD cells with kinase-dead murine IKKα(K44M)-BIN. Global views of micromass sphere sections (100Xmagnification) are shown on the left, and three random fields at 400Xmagnification submitted to quantitative image analysis are on the right. Antibody stained images are shown in the right side upper rows (labeled 1, 2 & 3), with each 400Xfield analyzed by Nikon Imaging Software in the lower rows (labeled 1.1, 2.1 & 3.1). For each sample an IHC staining threshold was established based on an antibody isotype control. Antibody staining is in red in conjunction with hematoxylin (blue) nuclear counterstaining. Data are representative examples of multiple sections of micromasses prepared with ACs of 3 OA patients; and results of all such experiments are presented as statistically analyzed bar graphs in Figure 7D.

**Figure 11:**
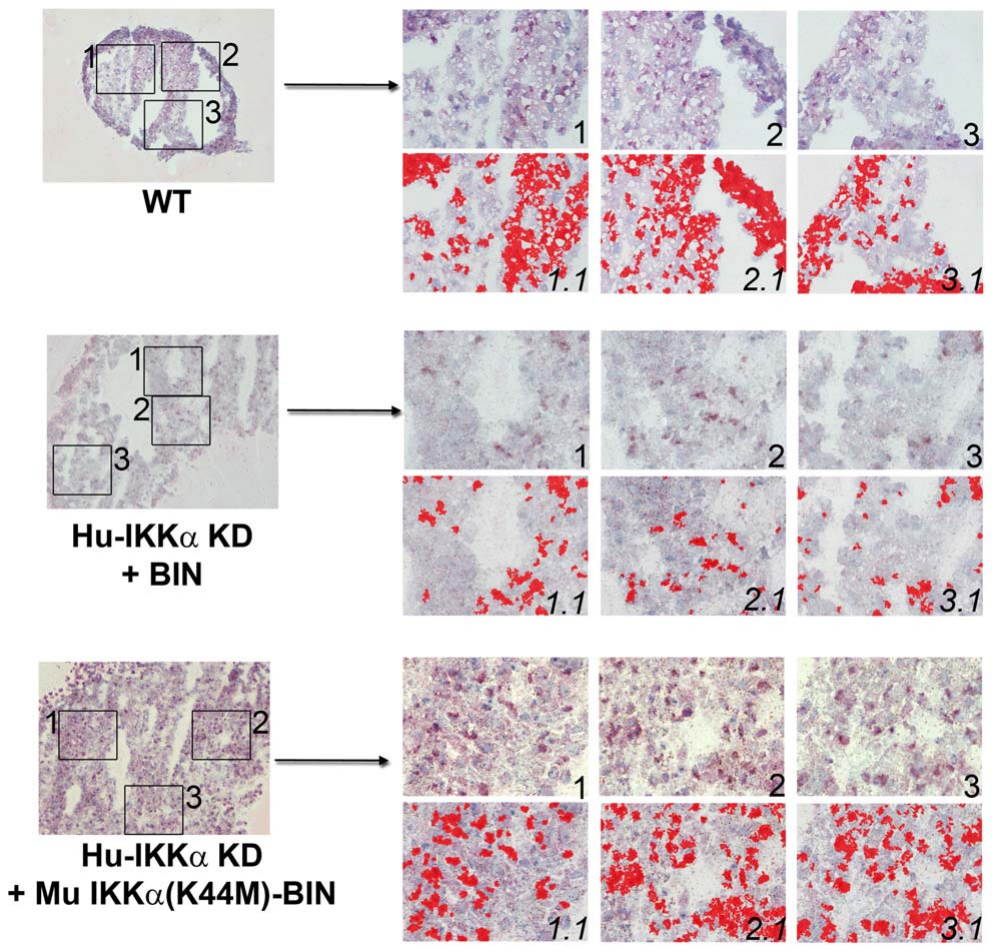
Effects of IKKα knock-down on Runx2 protein levels in human OA chondrocyte micromass cultures are independent of its kinase activity. Runx2 immunostaining in 3-week pellet cultures of primary human OA chondrocytes: WT (GL2 control), IKKα KD cells with empty BIN retroviral vector and IKKα KD cells with kinase-dead murine IKKα(K44M)-BIN. Global views of micromass sphere sections (100Xmagnification) are shown on the left, and three random fields at 400Xmagnification submitted to quantitative image analysis are on the right. Antibody stained images are shown in the right side upper rows (labeled 1, 2 & 3), with each 400Xfield analyzed by Nikon Imaging Software in the lower rows (labeled 1.1, 2.1 & 3.1). For each sample an IHC staining threshold was established based on an antibody isotype control. Antibody staining is in red in conjunction with hematoxylin (blue) nuclear counterstaining. Data are representative examples of multiple sections of micromasses prepared with ACs of 3 OA patients; and results of all such experiments are presented as statistically analyzed bar graphs in Figure 7E.

**Figure 12:**
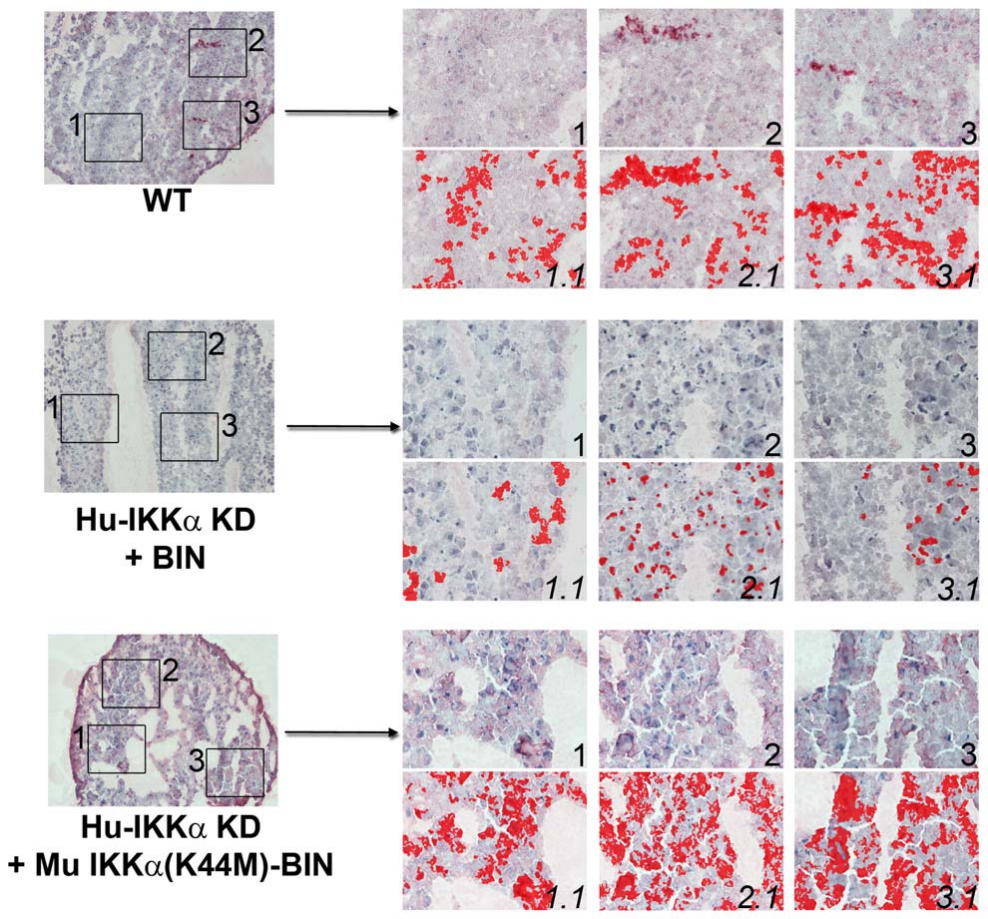
Effects of IKKα knock-down on COL10 protein levels in human OA chondrocyte micromass cultures are independent of its kinase activity. COL10 immunostaining in 3-week pellet cultures of primary human OA chondrocytes: WT (GL2 control), IKKα KD cells with empty BIN retroviral vector and IKKα KD cells with kinase-dead murine IKKα(K44M)-BIN. Global views of micromass sphere sections (100Xmagnification) are shown on the left, and three random fields at 400Xmagnification submitted to quantitative image analysis are on the right. Antibody stained images are shown in the right side upper rows (labeled 1, 2 & 3), with each 400Xfield analyzed by Nikon Imaging Software in the lower rows (labeled 1.1, 2.1 & 3.1). For each sample an IHC staining threshold was established based on an antibody isotype control. Antibody staining is in red in conjunction with hematoxylin (blue) nuclear counterstaining. Data are representative examples of multiple sections of micromasses prepared with ACs of 3 OA patients; and results of all such experiments are presented as statistically analyzed bar graphs in Figure 7E.

Next, we performed a series of experiments to determine if the kinase activity of IKKα was required for its functional effects in differentiating human OA chondrocytes and iMACs. To this end, we stably transduced endogenous IKKα-deficient (IKKα KD) human OA chondrocytes with retroviral vectors harboring epitope-tagged forms of either murine WT IKKα or a kinase-dead IKKα(K44M) recombinant mutant [72], [77]. Our IKKα KD human chondrocytes were generated by stable transduction with a puromycin resistance retroviral vector co-expressing a human IKKα-targeted shRNA, as described in our earlier work [40]. Thus, we stably introduced WT or mutant forms of murine IKKα with neomycin-resistant retroviral vectors in which the IKKα ORFs are co-expressed together with a *Neo* gene in a bi-cistronic expression cassette (BIN retroviral vectors) [70], [71] (Figure 7). Figure 7A shows immunoblots verifying the expression of the exogenous murine IKKα proteins in endogenous IKKα KD human OA chondrocytes. Enforced expression of exogenous WT murine IKKα or murine IKKα(K44M) proteins in human OA chondrocytes cultured for 1 week in differentiating pellets significantly reduced COL2 deposition in comparison to matched IKKα KD+empty BIN vector control cells, as revealed by *in situ* quantitative IHC imaging analysis (Figures 7B and 8). In analogous pellet cultures, the kinase-dead IKKα(K44M) mutant rescued the levels of MMP-13-dependent COL2–3/4C cleavage products almost up to their amounts in endogenous IKKα WT control cells (Figures 7C and 9). In addition, enforced expression of the IKKα(K44M) mutant reduced the enhanced levels of TIMP-3 protein in IKKα KD cells to their levels in WT control cells (Figures 7D and 10). Moreover, RUNX2 and COL10, markers of chondrocyte differentiation towards a hypertrophic-like state, also displayed comparable protein levels in WT and IKKα(K44M)-rescued cells (Figures 7E, 11 and 12).

**Figure 13:**
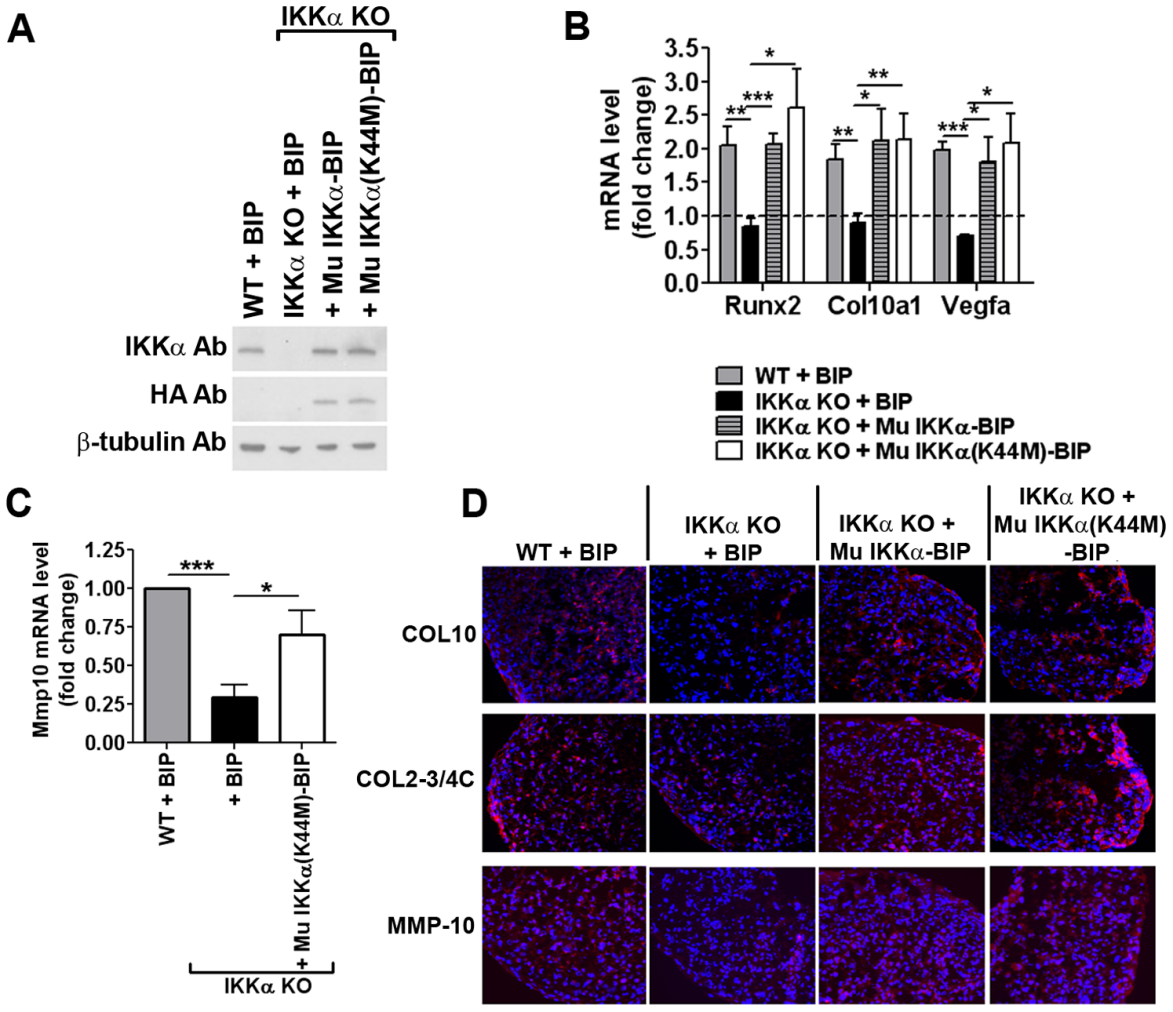
IKKα positively regulates iMAC ECM remodeling and differentiation independent of its kinase activity. (**A**)**:** Representative immunoblots of IKKα^f/f^;CreERT2 iMACs treated with EtOH (WT control) or 4-OHT (IKKα KO) and then stably transduced with a retroviral vector conferring puromycin resistance (BIP) compared to the same 4-OHT-treated IKKα KO cells transduced with BIP retroviral vectors co-expressing WT murine IKKα (Mu IKKα-BIP) or a murine IKKα kinase-dead mutant {Mu IKKα(K44M)}. Immunoblots were first probed with anti-IKKα antibodies and then sequentially re-probed with anti-HA and β-tubulin specific antibodies. (**B**) qRT-PCR analysis of Runx2, Col10a1 and Vegfa mRNA levels in 2-week iMAC high density monolayer cultures. As in Panel A, WT Mu IKKα-BIP or Mu IKKα(K44M)-BIP were transduced into 4-OHT-induced IKKα KO cells. Data are represented as fold-change vs. 1-week samples set at 1.0 (dotted line) and shown as mean ± S.E.M. (error bars). *p<0.05, **p<0.01 and ***p<0.001 by Student’s t-test. (**C**) qRT-PCR analysis of Mmp10 mRNA in 1-week monolayer cultures, with IKKα(K44M)-BIP in 4-OHT induced IKKα KO iMACs as indicated above. Data are shown as mean ± S.E.M. (error bars) derived from multiple experiments performed in duplicate and represented as fold-change vs. WT (EtOH treated) +BIP control. *p<0.05 and ***p<0.001 by Student’s t-test. (**D**) Immunostaining with COL10, C1,2C and MMP-10 specific antibodies of 1-week iMAC pellet cultures under the indicated conditions (Original magnification, 200X). Images are representative of 3 experiments.

In independent experiments, we stably transduced IKKα-ablated iMACs with BIP retroviral vectors that co-express either WT IKKα or the IKKα(K44M) mutant together with a puromycin resistance gene in a bi-cistronic expression cassette [70]–[72] (Figure 13). We employed puromycin-resistant retroviral vectors with iMACs because, unlike neomycin drug selection that requires up to 2 weeks to obtain resistant cell populations, the selection of puromycin-resistant cells only requires 2 to 3 cell passages during less than 1 week, thereby avoiding any concomitant effects due to additional cell doublings resulting in premature cell ageing. The efficacy and reproducibility of IKKα protein rescue was verified by immunoblotting (Figure 13A). Importantly, due to the significantly higher retroviral transduction frequencies of 4-OHT-induced IKKα KO iMACs, we employed diluted stocks of WT IKKα-BIP and IKKα(K44M)-BIP viruses to rescue IKKα protein levels to near physiological levels (Figure 13A), hence ruling out the possibility of IKKα over-expression artifacts. The analysis of total RNAs isolated from 2-week high density monolayer cultures showed that retroviral-mediated stable transduction of near physiological levels of murine WT IKKα or IKKα(K44M) into IKKα KO iMACs rescued the RNA expression levels of the hypertrophic differentiation markers Runx2, Col10a1 and Vegfa to the amounts detected in WT control iMACs transduced with a BIP empty vector (Figure 13B). Importantly, Mmp10 mRNA levels were also rescued to near physiological levels by the kinase-dead IKKα(K44M) protein (Figure 13C). Moreover, in accord with these results, immunohistochemical analysis of 1-week iMAC pellet cultures showed that the *in situ* protein levels of COL10, COL2-3/4C and MMP-10 were all effectively rescued by the kinase-dead IKKα(K44M) protein (Figure 13D).

## Discussion

### IKKα Modulates Chondrocyte ECM Remodeling by Transcriptional and Post-Transcriptional Mechanisms

As elaborated above in the Introduction, our earlier published work suggested that IKKα acts as a positive effector of AC differentiation by facilitating type II collagen remodeling [39], [40], and here, we have investigated mechanism of action of IKKα in this cellular context. In addition to using primary human articular OA chondrocytes, we employed inducible IKKα KO primary chondrocytes derived from 5- to 6-day-old *IKKα^f/f^;CreRT2* mice. We observed that the effects of IKKα on ECM remodeling and subsequent aspects of chondrocyte differentiation towards a hypertrophic-like state are evolutionarily conserved between human OA and murine ACs, and thus the functional effects of IKKα in chondrocytes are not linked to unique properties of cells originating from OA diseased cartilage (Figure 1 and Figure S1). We initially employed whole genome mRNA expression profiling as an unbiased screen for IKKα targets in differentiating human OA-derived ACs (Figure S2). We discovered that the *MMP10* (stromelysin-2) gene, which encodes a pro-collagenase activating protease [37], was a potential target of IKKα and subsequent qRT-PCR analysis validated that MMP10 mRNA was indeed an IKKα target in both human and murine ACs (Figure 2); and MMP10 protein levels were comparably suppressed in IKKα deficient cells (Figures 2 and 3). Additional experiments revealed that the levels of TIMP-3 protein, an inhibitor of MMPs and aggrecanases in chondrocytes, were also dependent on IKKα (Figures 4 and 5). However, unlike MMP-10, TIMP-3 protein levels were controlled by IKKα at the post-transcriptional level, because IKKα KD enhanced the accumulation of TIMP-3 protein with no effect on TIMP3 mRNA levels (Figure 4). In accord with these collective findings, we found that maximal MMP-13 and total collagenase activities in differentiating cultures of ACs were dependent on IKKα (Figure 6).

### IKKα’s Positive Effects on Chondrocyte ECM Remodeling and Differentiation are Independent of its Kinase Activity

To determine if the functional effects of IKKα in chondrocytes required its serine-threonine kinase activity, we rescued its expression in both IKKα KD human OA ACs and in IKKα KO iMACs by stable retroviral transduction with murine WT IKKα or a recombinant IKKα kinase-dead mutant derivative {IKKα(K44M)}. Detailed analyses of ECM status and differentiation potentials revealed that the enforced expression of an exogenous kinase-dead IKKα mutant protein in endogenous IKKα-deficient chondrocytes (of either human or murine origin) rescued type II collagen remodeling and restored the expression of a variety of chondrocyte differentiation markers including Col10, Runx2 and Vegfa (Figures 7–13). Importantly, the IKKα(K44M) mutant reversed the accumulation of TIMP-3 in IKKα KD human OA chondrocytes (Figure 7D and 10) and rescued the expression of Mmp10 mRNA and protein in IKKα KO iMACs (Figure 13C&D). Thus, we believe that our collective findings definitively show that IKKα facilitates MMP-dependent ECM remodeling and subsequent chondrocyte differentiation towards hypertrophy independent of its activity as a serine-threonine kinase. Future work will be necessary to precisely define how IKKα without its kinase activity acts to control the levels of upstream effectors of collagenases to facilitate ECM remodeling, which subsequently drives chondrocyte differentiation towards a hypertrophic-like phenotype. Indeed, a causative relationship between MMP-derived collagen fragments that accumulate during the onset of ECM remodeling and subsequent chondrocyte differentiation towards hypertrophy has been documented [12], [78]; and we have previously shown that, similar to IKKα loss, targeted MMP-13 mRNA ablation in human OA ACs also blocks their *in vitro* differentiation [39]. In addition, our prior published results suggested that IKKα or MMP-13 loss in ACs elicited Sox9-mediated inhibition of nuclear β-catenin activity [39], which was previously found necessary for chondrocyte terminal differentiation towards hypertrophy [42], thereby hinting of a mechanistic link between the positive effects of IKKα or MMP-13-mediated ECM remodeling and subsequent steps in chondrocyte differentiation.

### On the Relevance of Functional Effects of IKKα in Chondrocyte ECM Remodeling and Differentiation in the Context of OA Disease Development

The importance of IKKα in ECM remodeling (analogous to that of MMP-13) may only come into play when growth-arrested ACs are coaxed to differentiate towards a hypertrophic-like state. Aspects of this hypertrophic-like differentiation are recapitulated by articular chondrocytes (ACs) *in vitro*, using models of endochondral ossification [40], and *in vivo* under the exacerbated stress associated with the onset and progression of OA disease. Indeed, the loss of normal cartilage homeostasis in OA disease resembles to varying degrees the conversion of periarticular chondrocytes to a differentiated, hypertrophic-like state, and this process is associated with the inappropriate expression and activation of MMP-13 {reviewed in [30], [48]}. Although MMP-13 KO mice thrive throughout adulthood with normal cartilage formation and bone growth aside from somewhat enlarged hypertrophic zones in the joints of new born mice, which are eventually resolved by adulthood [28], [79], MMP-13 deficiency in mice is protective against cartilage erosion that develops in surgically induced OA disease [27], [29]. Thus, we posit that in the context of murine embryonic development, and also consistent with the properties of MMP-13 *in vivo*, IKKα may not have an essential (e.g., irreplaceable) function in ECM remodeling and subsequent chondrocyte differentiation if its functional effects overlap with other regulatory factors during embryonic or post-natal development *in vivo,* and/or if the functional impact threshold of IKKα on ECM remodeling is insufficient to perturb the extent of terminal chondrocyte differentiation required for endochondral ossification in mammalian embryogenesis. Future work will be necessary to determine if the induced *in vivo* ablation of IKKα in the ACs of adult mice has protective effects against the onset or advanced progression of acute surgically induced OA disease.

## Supporting Information

Figure S1 **Effects of IKKα ablation on iMAC matrix production and remodeling.** **(A, upper)** Additional representative type II collagen (COL2) immunofluorescence staining of 3-week high density monolayer cultures of IKKα^f/f^;CreERT2 iMACs treated with EtOH/vehicle (WT) or 4-OHT (IKKα KO). Merged images are shown. Green: COL2; Blue: DAPI. **(A, lower)** Representative COL2 immunohistochemical staining of 3-week pellet cultures of WT and IKKα KO IKKα^f/f^;CreERT2 iMACs. **(B)** Additional representative COL2–3/4C immunohistochemical staining of 3-week pellet cultures of WT and IKKα KO IKKα^f/f^;CreERT2 iMACs. Merged images are shown. Red: COL2–3/4C; Blue: DAPI. All images in **A** and **B** are 100Xmagnifications of originals.
(TIF)

Figure S2 **Microarray gene expression profiling of wild-type GL2 Control vs. IKKα KD (knock-down) differentiating human OA articular chondrocyte micromass cultures.** Hierarchical clustering image comparing the differential expression profiles of 100 genes (p<0.01), which were most affected by IKKα KD (lanes A2, A3, A4) compared to wild-type (WT) control GL2 (C2, C3, C4 lanes) in differentiating 1-week micromass cultures derived from the articular chondrocytes (ACs) of multiple human OA patients (N of 3). Relative expression levels are shown in Log2 scale format with induced and repressed genes in red and green, respectively. Note that MMP10, a collagenase activator, is strongly repressed in the IKKα KD cells and its reduced expression level also co-clusters with that of IKKα.
(TIF)
