# Supplementary figures and images for: IKKα/CHUK Regulates Extracellular Matrix Remodeling Independent of Its Kinase Activity to Facilitate Articular Chondrocyte Differentiation
Source: PLoS One. 2013 Sep 2;8(9):e73024. doi: 10.1371/journal.pone.0073024 (PMC3759388; doi:10.1371/journal.pone.0073024)

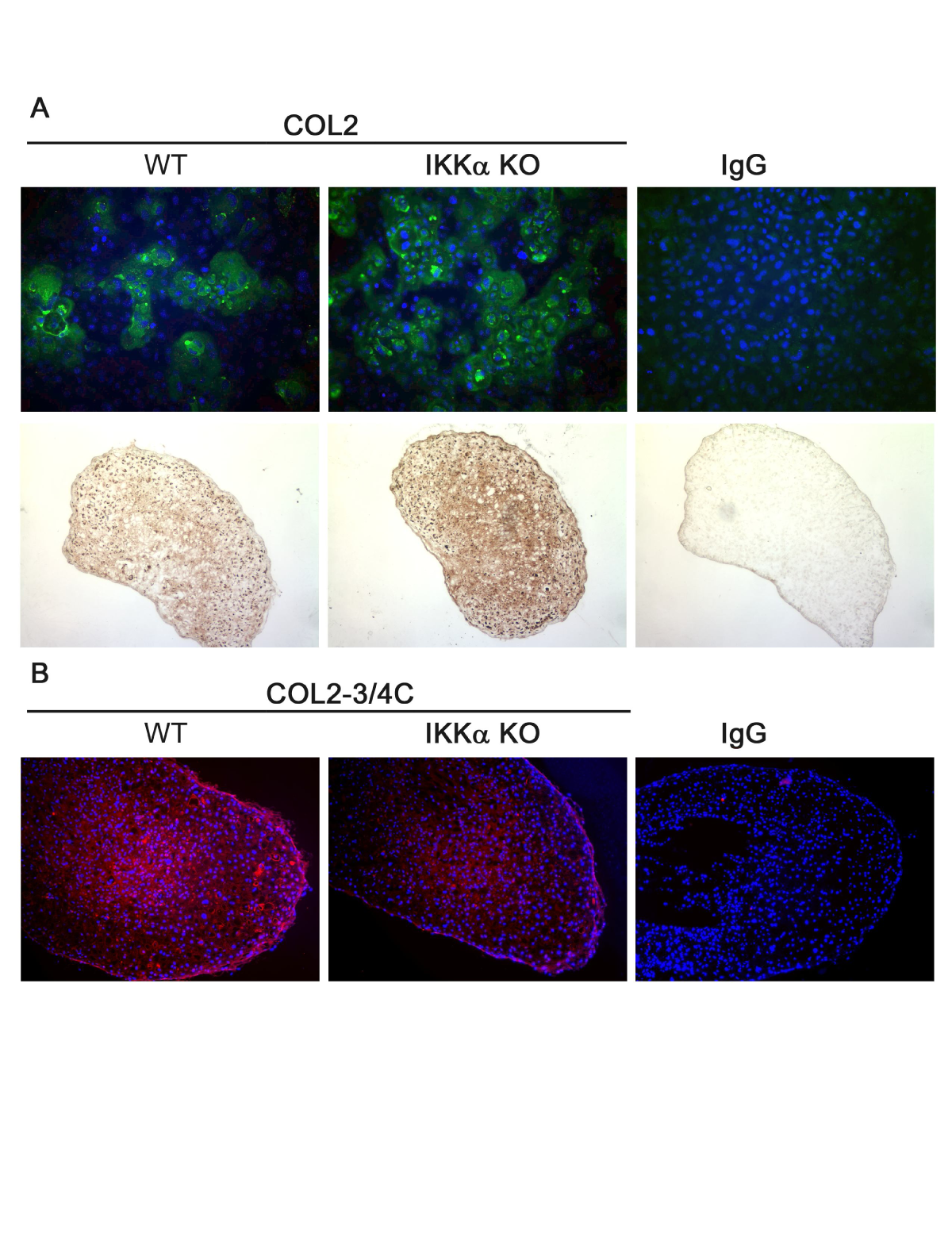

Supplement: Figure S1 — Effects of IKKα ablation on iMAC matrix production and remodeling. (A, upper) Additional representative type II collagen (COL2) immunofluorescence staining of 3-week high density monolayer cultures of IKKαf/f;CreERT2 iMACs treated with EtOH/vehicle (WT) or 4-OHT (IKKα KO). Merged images are shown. Green: COL2; Blue: DAPI. (A, lower) Representative COL2 immunohistochemical staining of 3-week pellet cultures of WT and IKKα KO IKKαf/f;CreERT2 iMACs. (B) Additional representative COL2–3/4C immunohistochemical staining of 3-week pellet cultures of WT and IKKα KO IKKαf/f;CreERT2 iMACs. Merged images are shown. Red: COL2–3/4C; Blue: DAPI. All images in A and B are 100Xmagnifications of originals. (TIF) [file pone.0073024.s001.tif]

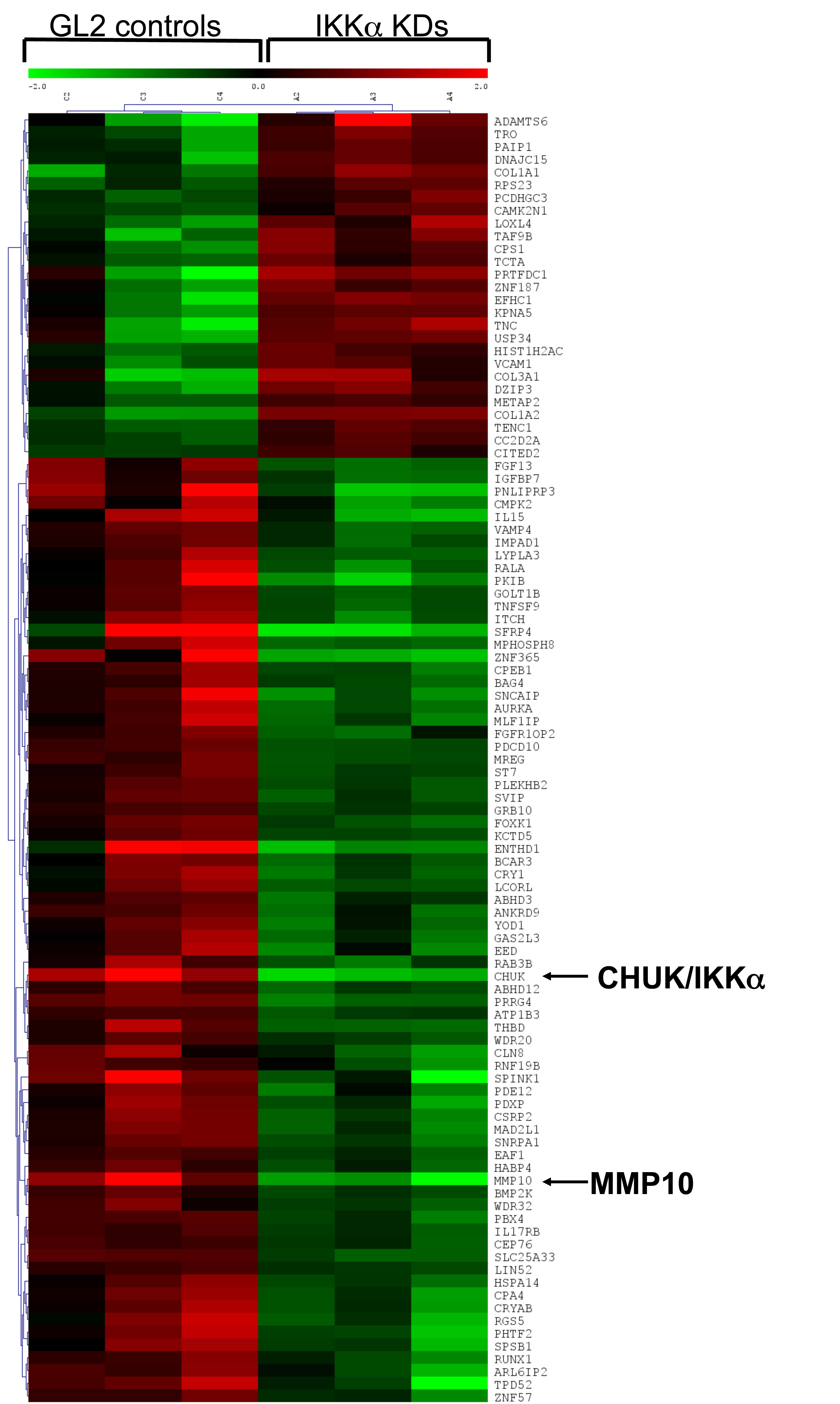

Supplement: Figure S2 — Microarray gene expression profiling of wild-type GL2 Control vs. IKKα KD (knock-down) differentiating human OA articular chondrocyte micromass cultures. Hierarchical clustering image comparing the differential expression profiles of 100 genes (p<0.01), which were most affected by IKKα KD (lanes A2, A3, A4) compared to wild-type (WT) control GL2 (C2, C3, C4 lanes) in differentiating 1-week micromass cultures derived from the articular chondrocytes (ACs) of multiple human OA patients (N of 3). Relative expression levels are shown in Log2 scale format with induced and repressed genes in red and green, respectively. Note that MMP10, a collagenase activator, is strongly repressed in the IKKα KD cells and its reduced expression level also co-clusters with that of IKKα. (TIF) [file pone.0073024.s002.tif]
